# HOME-BASED DIGITAL EXERCISE VERSUS HOSPITAL PHYSIOTHERAPY FOR PLANTAR FASCIITIS: A PROPENSITY-SCORE-MATCHED RETROSPECTIVE COHORT STUDY WITH DUAL-PERSPECTIVE COST-UTILITY EVALUATION

**DOI:** 10.2340/jrm.v58.45974

**Published:** 2026-09-02

**Authors:** Wenbo XU, Lufeng YAO, Haigang WANG, Chenqin XU, Pinpin HE, Yi ZHANG, Jiasong PAN, Shengdi LU, Lei HUANG

**Affiliations:** 1 Department of Foot and Ankle , Ningbo No.6 Hospital, Ningbo, Zhejiang, China; 2 Ningbo Clinical Research Center for Orthopedics, Sports Medicine & Rehabilitation, Ningbo, Zhejiang, China; 3 Department of Rehabilitation Medicine, Ningbo No. 6 Hospital, Ningbo, Zhejiang, China; 4 Pennington Biomedical Research Center, Baton Rouge, LA, USA

**Keywords:** plantar fasciitis, digital therapeutics, telerehabilitation, propensity-score matching, cost-utility analysis

## Abstract

**Objective:**

To compare the clinical effectiveness and cost-effectiveness of a 12-week home-based digital exercise programme with 12-week hospital-supervised physiotherapy for plantar fasciitis in routine Chinese tertiary-hospital care.

**Design:**

Single-centre retrospective cohort study with 1:2 propensity-score matching on 13 baseline covariates.

**Subjects/Patients:**

587 adults with a clinician-confirmed diagnosis of plantar fasciitis treated in 2022–2024 were matched.

**Methods:**

*T*he primary outcome was first-step morning pain at 3 months on a 0–10 numeric rating scale (non-inferiority margin 1.3 points; sensitivity margins 0.9 and 1.9). A 12-month cost–utility analysis took payer and societal perspectives.

**Results:**

The adjusted mean difference was −0.63 points (95% confidence interval −0.92 to −0.35; *p* < 0.001), non-inferior against all 3 margins (E-value 2.84). Incremental payer cost was −¥4,920 (−US$684), incremental societal cost −¥7,008 (−US$974), and incremental quality-adjusted life-years +0.011, with dominance in 96.7% of bootstrap replications.

**Conclusion:**

Home-based digital exercise was non-inferior for 3-month first-step pain and less costly from both perspectives. Matching left 12 of 13 covariates imbalanced and only 6.3% of screened patients were eligible, so residual confounding and selection bias are likely. These observational findings are provisional and require confirmation in a pragmatic multicentre randomized controlled trial before informing reimbursement.

## LAY ABSTRACT

Plantar fasciitis causes heel pain that is worst on the first steps of the morning. It is usually treated with exercises, which in China are often taught during repeated hospital visits costing time and money. We reviewed the records of 587 adults treated at one Chinese hospital from 2022 to 2024. Some followed a 12-week exercise programme at home using a smartphone app; others attended hospital physiotherapy. We compared 384 patients who were similar to one another. After 3 months, morning heel pain had improved at least as much in the app group, at lower cost to both the health insurer and society. This is the first such comparison in China, and suggests app-based care could widen access where therapists are scarce. Because patients chose their own treatment, the 2 groups still differed in important ways. A randomized study is needed before app-based care replaces hospital physiotherapy.

Plantar fasciitis (PF) is common and costly. It accounts for most clinical presentations of heel pain in ambulatory care, and up to 1 in 10 adults experience it in a lifetime (1–3). In the United States, PF drives nearly 1 million physician visits each year and imposes direct costs on third-party payers estimated at US$192 to US$376 million annually (4, 5). A recent Korean national insurance sample reports a comparable per-patient burden (6). Chinese population-level prevalence estimates are not yet available. Rehabilitation demand in China is nonetheless substantial: recent burden-of-disease work indicates more than 460 million Chinese residents need rehabilitation services, against only 1.16 rehabilitation physicians per 100,000 population (7, 8).

First-line management is conservative. Clinical Practice Guidelines (CPGs) endorse structured exercise as the cornerstone of care for at least 6 to 12 weeks before any escalation. Core components are plantar fascia-specific stretching, progressive calf-and-foot strengthening, and education on load management. The 2023 American Physical Therapy Association (APTA) Heel Pain CPG gives these interventions Grade A recommendations (9, 10). Delivering such a programme through twice- or thrice-weekly hospital physiotherapy is demanding in Chinese routine care. Travel times are long. Co-payments add up. Scheduling capacity is limited by the meagre specialist workforce (7, 8).

Digital health offers a plausible alternative. By December 2024, China had 1.108 billion internet users and 78.6% mobile penetration (11, 12). App-delivered exercise programmes for musculoskeletal conditions have achieved outcomes comparable to in-person physical therapy in randomized trials of knee pain and knee osteoarthritis (13–17). The 2024 PEAK trial demonstrated non-inferiority of video-based telerehabilitation vs in-person consultations for chronic knee pain (15). A digital programme may further add structured progression, automated adherence prompts, and remote therapist oversight beyond what a printed home-exercise leaflet provides. Whether these advantages extend to PF, to Chinese patients, and to an asynchronous app-delivered model remains unclear. To our knowledge, no published real-world study has compared a home-based digital exercise programme against hospital-supervised physiotherapy for PF in a Chinese population.

Non-inferiority framing is appropriate here. Digital home exercise is unlikely to out-perform supervised in-person care on every axis; its value proposition is comparable effectiveness at substantially lower cost and greater geographic reach. Evidence must therefore rule out clinically meaningful inferiority. Real-world comparative studies can supply such evidence, if designed with care to address confounding by indication.

We conducted a retrospective propensity-score-matched cohort study at a single Chinese tertiary hospital. We compared the Joymotion^®^ 12-week home-based digital exercise programme (Joymotion; Shanghai Fudong Medical Management Co., Ltd., Shanghai, China) against 12-week hospital-supervised physiotherapy in adults with PF.) 12-week home-based digital exercise programme against 12-week hospital-supervised physiotherapy in adults with PF. The aim was to estimate the comparative clinical effectiveness and cost-effectiveness of Joymotion^®^ digital home exercise vs hospital-supervised physiotherapy for PF over 12 months, using the first-step morning numeric rating scale (NRS) pain score at 3 months as the primary outcome. Two hypotheses were prespecified. First, the digital programme would be non-inferior to hospital-supervised physiotherapy on 3-month first-step morning NRS pain, with a non-inferiority margin of 1.3 points derived from the Landorf minimal important difference (MID) framework and triangulated at 0.9 and 1.9 points in sensitivity analyses. Second, the digital programme would dominate hospital supervision, being both less costly and at least as effective, from payer and societal perspectives at the Chinese willingness-to-pay (WTP) threshold of ¥157,300 per quality-adjusted life-year (QALY).

## METHODS

### Study design and setting

We conducted a single-centre retrospective cohort study with propensity score (PS) matching at a tertiary teaching hospital in eastern China. The hospital serves an urban and peri-urban catchment of approximately 4.2 million residents, with mixed insurance coverage under the Urban Employee and Urban-Rural Resident Basic Medical Insurance schemes. The study window spanned 1 January 2022 to 31 December 2024, encompassing the late-pandemic lockdown phase, the national reopening transition around January 2023, and the post-reopening period. Reporting follows the Strengthening the Reporting of Observational Studies in Epidemiology (STROBE) statement (18) and the Consolidated Health Economic Evaluation Reporting Standards 2022 (CHEERS 2022) statement (19). The analysis plan (including the primary non-inferiority margin) was finalized before data extraction. The protocol was approved by the institutional ethics committee, which waived informed consent for de-identified electronic health record (EHR) data.

### Participants

Eligible patients were ≥ 18 years with a clinician-confirmed primary diagnosis of PF coded M72.2 under the Chinese national disease classification (GB/T 14396-2016; National Clinical Version 2.0) (20), consistent with the APTA 2023 CPG diagnostic criteria of insidious, load-related medial plantar heel pain worst on the first step after inactivity, with reproducible tenderness at the medial calcaneal tubercle (9). We excluded patients with secondary plantar heel pain, prior plantar fascia surgery, incomplete 12-week clinical records, or missing baseline first-step NRS. Plantar fascia thickness ≥ 4.0 mm on ultrasound supported diagnosis when available (21, 22).

### Interventions

Allocation to the 2 pathways reflected patient preference, geographic proximity to the clinic, and scheduling capacity rather than randomization. The Joymotion® home-based digital programme delivered a 12-week progressive exercise package through a smartphone app with video instruction, weekly asynchronous therapist check-ins (typically 10 min per patient per week), adherence logs, and symptom-triggered escalation rules (23). The hospital-supervised programme delivered the same 12-week exercise framework in 2 to 3 face-to-face sessions per week with added manual therapy and clinic-based modalities. Both groups used identical progression anchors: pain relief and stretching in weeks 1–2; progressive loading in weeks 3–4; high-load calf-and-foot strengthening per Rathleff in weeks 5–6; functional loading in weeks 7–8; sport-specific return in weeks 9–10; and maintenance in weeks 11–12 (24–27). The full week-by-week protocol, baseline tailoring rules, and in-programme regulation algorithm are in Tables SX–SXII. Programme tariffs were ¥1,980 (digital) and ¥5,760 (hospital); therapist asynchronous-review time in the digital group was included in the programme tariff and in the payer-perspective cost inventory.

### Outcomes

The primary outcome was the first-step morning NRS pain score (0–10) at 3 months. The non-inferiority margin of 1.3 points was prespecified by reference to the Landorf MID framework for plantar heel pain (28). Landorf and colleagues reported anchor-based MIDs of approximately 0.8 points on a 0–10 scale for average pain and approximately 1.9 points for first-step pain; 1.3 points was chosen as the midpoint between these 2 anchors, more conservative than the first-step MID and less conservative than the average-pain MID. We report the primary non-inferiority conclusion against alternative margins of 0.9 and 1.9 points to demonstrate robustness to margin choice.

Secondary outcomes were resting and activity NRS; the Foot and Ankle Ability Measure (FAAM) Activities of Daily Living and Sport subscales (29); the American Orthopedic Foot and Ankle Society (AOFAS) ankle–hindfoot score; EuroQol 5-dimension 5-level (EQ-5D-5L) utility derived from the Luo 2017 Chinese value set (30); EQ-5D visual analogue scale; the Work Productivity and Activity Impairment (WPAI) score; the proportion achieving the minimal clinically important difference (MCID) of ≥ 1.3 NRS; 12-month symptomatic recurrence; and conversion to surgery. All secondary outcomes are presented with nominal 95% confidence intervals (CIs) and are hypothesis-generating rather than confirmatory.

### Data sources and measurement

EHR variables were extracted through a structured query run against the hospital’s clinical data warehouse, with dual independent review of 10% of charts (κ = 0.91; intraclass correlation 0.96). The extraction dictionary is in Table SIX. The primary outcome and all NRS/PROM scores were collected through the hospital’s tablet-based intake station at each follow-up visit using a single harmonized form, with the same wording in both groups; the digital group did not report outcomes via the Joymotion app, eliminating 1 potential source of ascertainment asymmetry. Residual asymmetry (e.g., clinic context cueing in the hospital group) is addressed in the Bias subsection.

### Bias

We mapped each threat to internal validity to a specific mitigation. Confounding by indication was addressed by 1:2 PS matching on 13 baseline covariates, mixed-effects regression adjustment within the matched cohort, inverse-probability-of-treatment weighting (IPTW) on the full cohort as a triangulation, and *E*-value quantification of robustness to any single unmeasured confounder (31–34). Digital-adoption confounders not directly captured in structured EHR fields, digital literacy, smartphone ownership duration, prior app use, self-efficacy, health literacy, and intrinsic motivation, are discussed as residual unmeasured confounding in the Limitations section. Selection bias from the eligibility requirement of a complete 12-week record was addressed by an IPTW sensitivity analysis on the full cohort including partial-record patients, recognizing that this requirement conditions the analytic sample on post-baseline information and is examined further as a primary limitation (46). Information bias from differential ascertainment was mitigated by using the same tablet-based PROM instrument in both groups and is further examined in a tipping-point bias analysis. Attrition bias was addressed by multiple imputation (MI) with chained equations (*m* = 20) under missing-at-random, and by pattern-mixture δ-shift sensitivity penalizing the digital group (35, 36). Immortal-time bias was not applicable because the 12-week intervention window was fixed and defined from the index visit. Calendar-time confounding by the COVID-19 reopening was addressed by including enrolment quarter as a covariate in the PS model and by a sensitivity analysis restricted to enrolments after 1 January 2023.

### Study size

The study used the full eligible cohort over the study window; the following calculation documents the power afforded by the realized sample rather than determining enrolment. For a non-inferiority comparison of a continuous outcome with 2 hospital patients matched to each digital patient (allocation ratio k = 2), the minimum number of digital patients is n = (1 + 1/k)(z_1_–α + z_1_–β)²σ²/Δ², with a corresponding hospital sample of k·n (49, 50). With a non-inferiority margin Δ = 1.3 points on the first-step NRS, an expected standard deviation (SD) σ = 2.4, an assumed true between-group difference of 0, a 1-sided α = 0.025 (z_1_–α = 1.960), and power 0.90 (z_1_–β = 1.282), the minimum matched sample was 54 digital and 108 hospital patients (162 in total). The realized matched sample (128 digital, 256 hospital) substantially exceeded this minimum. At the realized sample size, the study had approximately 99% power against the primary 1.3-point margin and approximately 93% power against the more conservative 0.9-point margin (Table SIII).

### Quantitative variables

Thirteen prespecified baseline covariates entered the PS model, selected *a priori* as potential common causes of programme assignment and outcome (39, 41). They comprised 4 sociodemographic and clinical factors (age, sex, body mass index [BMI], and the Charlson comorbidity index), 1 diagnosis-specific comorbidity (type-2 diabetes mellitus), 1 disease-history factor (prior PF episode), 1 baseline disease-severity measure (baseline first-step morning NRS), and 6 access- and digital-engagement factors that plausibly drove the choice between a home-based digital programme and hospital attendance (education at or above the tertiary level, urban residence, insurance type [Urban Employee Basic Medical Insurance, Urban-Rural Resident Basic Medical Insurance, or out-of-pocket], distance to the hospital, appointment made via the mobile app or WeChat, and an active e-prescription flag). Insurance type was modelled as a single categorical covariate with 3 levels. Continuous covariates with potentially non-linear associations (age, BMI, and distance to hospital) were entered as restricted cubic splines. The full covariate set, the fitted PS model, and pre- and post-match balance are reported in Table I and Tables SI–SII.

**Table I:** Baseline characteristics before and after propensity-score matching

Characteristic	Pre-match digital (*n* = 145)	Pre-match hospital (*n* = 442)	Pre SMD	Post-match digital (*n* = 128)	Post-match hospital (*n* = 256)	Post SMD
Age, years, mean (SD)	44.3 (9.3)	52.9 (9.3)	0.92	45.4 (8.8)	50.4 (9.1)	0.56
Female, *n* (%)	115 (79.3)	276 (62.4)	0.38	100 (78.1)	172 (67.2)	0.25
BMI, kg/m², mean (SD)	25.0 (3.3)	25.3 (3.5)	0.11	25.1 (3.3)	25.1 (3.6)	0.01
Education≥tertiary, *n* (%)	70 (56.5)	90 (22.3)	0.62	58 (53.2)	69 (29.1)	0.39
Urban residence, *n* (%)	143 (98.6)	332 (75.1)	0.74	126 (98.4)	220 (85.9)	0.48
Insurance: UEBMI, *n* (%)	104 (71.7)	225 (50.9)	0.44	89 (69.5)	143 (55.9)	0.29
Insurance: URRBMI, *n* (%)	40 (27.6)	208 (47.1)	0.41	38 (29.7)	109 (42.6)	0.27
Insurance: out-of-pocket/other, *n* (%)	1 (0.7)	9 (2.0)	0.12	1 (0.8)	4 (1.6)	0.07
Appointment via mobile app/WeChat, *n* (%)	107 (73.8)	129 (29.2)	1.00	93 (72.7)	120 (46.9)	0.54
E-prescription flag, *n* (%)	98 (67.6)	97 (21.9)	1.03	83 (64.8)	83 (32.4)	0.69
Distance to hospital, km, median (IQR)	6.7 (4.4–9.6)	8.2 (4.8–16.2)	0.40	6.7 (4.3–9.6)	7.2 (4.5–13.8)	0.28
Charlson comorbidity index, mean (SD)	0.05 (0.22)	0.21 (0.46)	0.47	0.05 (0.23)	0.16 (0.38)	0.35
Baseline morning NRS (0–10), mean (SD)	7.07 (0.49)	7.15 (0.51)	0.15	7.05 (0.48)	7.14 (0.54)	0.17
Prior PF episode, *n* (%)	34 (28.3)	73 (18.7)	0.23	30 (28.6)	49 (22.0)	0.15
Type-2 diabetes mellitus, *n* (%)	3 (2.1)	69 (15.6)	0.49	3 (2.3)	30 (11.7)	0.37

Values are *n* (%), mean (SD), or median (IQR). SMD: standardized mean difference (absolute value). UEBMI: Urban Employee Basic Medical Insurance; URRBMI: Urban-Rural Resident Basic Medical Insurance. Post-match 1:2 allocation: 128 digital:256 hospital. Pre-match *c*-statistic of the propensity-score model = 0.78. 17 digital and 186 hospital patients were unmatched; 114 caliper violations were recorded. All 13 covariates achieved |SMD| <0.10 post-match, meeting the pre-specified balance target.

### Statistical methods

PS were estimated by logistic regression and used in 1:2 greedy nearest-neighbour matching without replacement, with a caliper of 0.2 × SD of the logit (31–33). Balance was judged adequate at a standardized mean difference (SMD) below 0.10 for every covariate (34). The primary analysis estimated the between-group difference in the matched cohort using all available outcome data, without restricting to patients with complete follow-up: a mixed-effects linear model with fixed effects for treatment, baseline score, categorical follow-up time (3, 6, and 12 months), a treatment × time interaction, and the 13 baseline covariates, with nested random intercepts for patient and for matched set (patient nested within matched set). Missing values in the baseline covariates (4.9–14.6% per variable) were multiply imputed by chained equations (*m* = 20), the imputation model including treatment, all covariates, and all observed outcomes; estimates were pooled by Rubin’s rules. Missing outcome values were not imputed in the primary analysis and were handled by maximum likelihood under a missing-at-random assumption. The data are hierarchical at 3 levels, because repeated measurements at 3, 6 and 12 months are nested within patients, who are in turn nested within 1:2 matched sets. Specifying a matched-set random intercept alone would impose an exchangeable (compound-symmetry) correlation across every observation in a matched set, so that 1 patient’s 3-month value and a different patient’s 12-month value would be assumed as strongly correlated as 2 measurements on the same patient; this mis-specifies the covariance structure and yields standard errors, and therefore inference, that are not valid (51, 52). The nested specification separates within-patient correlation due to repeated measures from between-patient correlation induced by matching, while retaining the matched-set level preserves the pairing created by the design (53). Denominator degrees of freedom used the Kenward–Roger approximation, which is appropriate for the modest number of matched sets (54). Modelling time as a categorical factor placed no functional-form constraint on the shape of the recovery trajectory. As a confirmatory analysis that removes the repeated-measures structure altogether, we additionally fitted a matched-set analysis of covariance (ANCOVA) restricted to the 3-month primary endpoint, in which each patient contributes a single observation and the only remaining source of non-independence is matched-set clustering; this model adjusted for the baseline score and the 13 covariates with a matched-set random intercept and omitted the time factor and the treatment × time interaction (55). The 3-month first-step NRS was the sole primary endpoint and was tested at a 2-sided 95% confidence level (equivalently, 1-sided α = 0.025 for the non-inferiority comparison); because the primary inference rests on this single endpoint, no multiplicity adjustment was applied. Contrasts at 6 and 12 months and all secondary outcomes were pre-specified as exploratory and are reported with nominal 95% CIs. Non-inferiority was declared if the upper bound of the 2-sided 95% CI at 3 months lay below +1.3. Because all post-match SMDs were below the 0.10 threshold, outcome-model adjustment for the matching covariates was undertaken to provide a doubly robust estimate and to address any residual within-pair imbalance rather than out of necessity (48); a parsimonious model adjusting only for baseline score was pre-specified as a sensitivity analysis to assess the influence of the covariate set on the estimate and its generalizability. A model adding a random slope for time and a per-protocol (PP) analysis restricted to patients completing at least 80% of prescribed sessions were also pre-specified.

Robustness to unmeasured confounding was quantified with the *E*-value (37). Because the primary outcome is continuous, the VanderWeele–Ding continuous approximation RR≈exp(0.91 × d) was used, with Cohen’s *d* = adjusted mean difference/pooled baseline SD, and *E* = RR + √[RR × (RR − 1)]. The pooled baseline SD of 1.6 was used; *E*-values are also reported using the residual within-group SD of 1.1 as a sensitivity.

The economic evaluation adopted a dual (payer and societal) perspective over a 12-month horizon, without discounting given the short horizon, following CHEERS 2022 (19). QALYs were calculated by trapezoidal area-under-the-curve from EQ-5D-5L utilities measured at baseline, 4 weeks, 3, 6, and 12 months, using the Luo 2017 Chinese tariff (30). Direct costs were micro-costed from EHR billing, including therapist asynchronous-review time in the digital group. Indirect costs used WPAI-derived absenteeism and presenteeism, valued at provincial mean wage. Probabilistic sensitivity analysis used 1,000 non-parametric bootstrap replications with matched-pair cluster resampling and a fixed seed. Cost-effectiveness was benchmarked against the Chinese WTP threshold of ¥157,300 per QALY (1.76 × 2023 GDP per capita) (38), with cost-effectiveness acceptability curves at 1× GDP and 3× GDP as sensitivity. Analyses used R 4.3.2 with MatchIt, lme4, lmerTest, pbkrtest, mice, boot, *E*-value, and cobalt packages; nested random effects were fitted in lme4 and Kenward–Roger degrees of freedom obtained via pbkrtest.

### Additional sensitivity analyses

Pre-specified sensitivity analyses were: (*i*) full-cohort regression adjustment; (*ii*) IPTW on the full cohort; (*iii*) a complete-case analysis restricted to patients with an observed 3-month outcome, conducted as a sensitivity analysis only and not as the primary analysis; (*iv*) MI with chained equations, in which missing post-baseline outcome values (3-, 6-, and 12-month NRS and patient-reported outcomes for unattended visits) were imputed for all matched patients and results pooled by Rubin’s rules; (*v*) pattern-mixture δ-shift with δ ∈ {0.5, 1.0, 1.5, 2.0} NRS; (*vi*) calendar-time sensitivity restricted to enrolments after January 2023; (*vii*) non-inferiority conclusion at alternative margins of 0.9 and 1.9 points; (*viii*) tipping-point analysis for differential outcome measurement; (*ix*) a mixed-effects model adding a patient-level random slope for time within the nested random-effects structure; (*x*) a parsimonious model adjusting only for baseline score; and (*xi*) a matched-set ANCOVA restricted to the 3-month primary endpoint, in which each patient contributes a single observation.

## RESULTS

### Participant flow and baseline characteristics

**Fig. 1:**
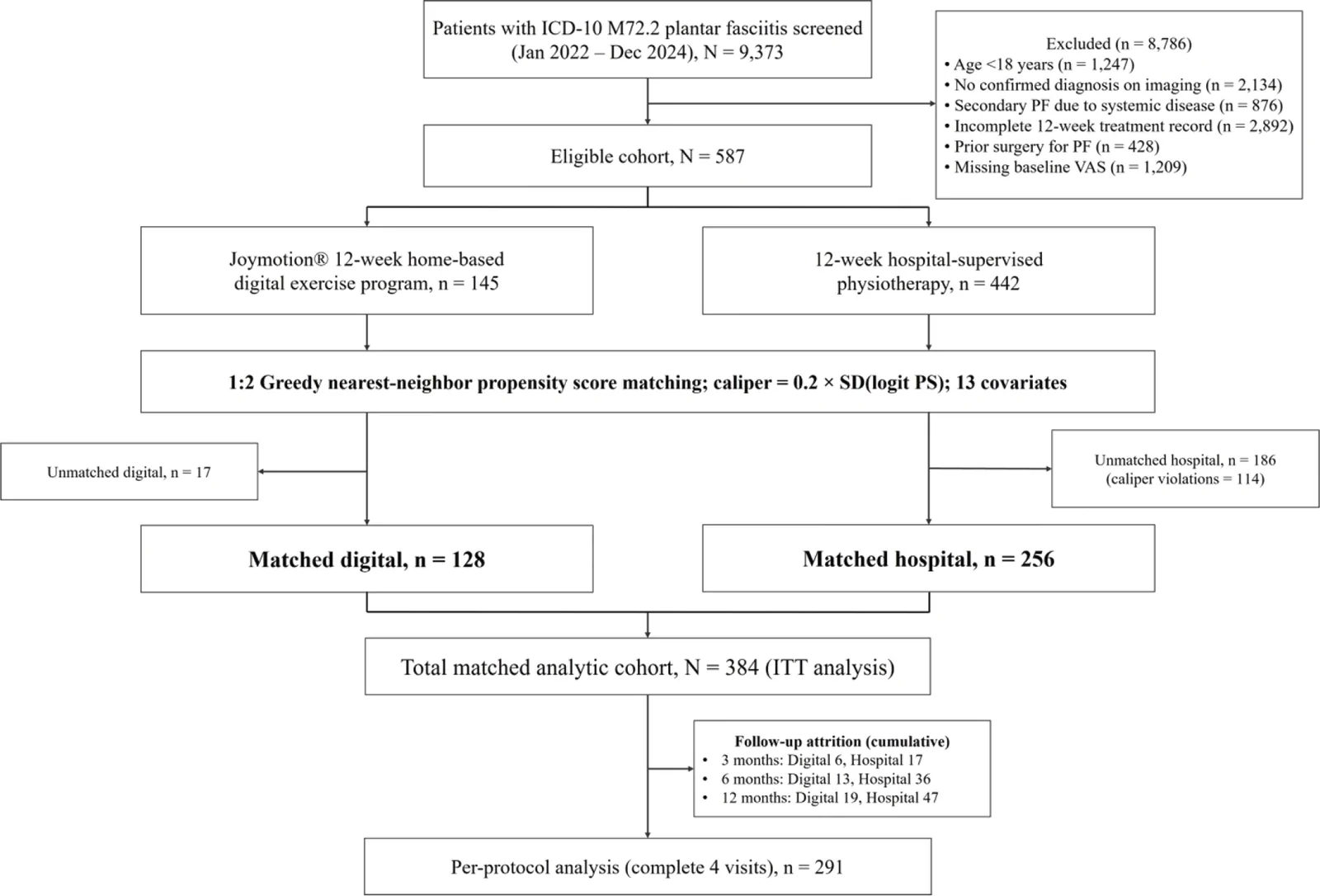
STROBE participant flow diagram. STROBE-compliant flow of participants through the retrospective cohort. Of 9,373 patients with ICD-10 M72.2 screened between January 2022 and December 2024, 587 were eligible: 145 received the Joymotion® 12-week home-based digital exercise programme and 442 received 12-week hospital-supervised physiotherapy. 1:2 greedy nearest-neighbour PS matching with a caliper of 0.2 × SD of the logit, applied to 13 prespecified baseline covariates, produced the matched analytic cohort (*N* = 384; 128 digital and 256 hospital patients). The per-protocol subset included 291 participants who completed all 4 follow-up visits. Cumulative attrition is reported at each time-point.

Of 587 screened patients, 145 entered the digital group and 442 the hospital group; reasons for exclusion are summarized in Fig. 1. After 1:2 PS matching, 384 patients (128 digital, 256 hospital) formed the analytic cohort. Seventeen digital patients (11.7%) and 186 hospital patients had no matched counterpart within caliper. Because 1:2 matching retains the hospital patients who most closely resemble the digital patients, the matched hospital group shifted towards the digital covariate profile relative to the full hospital group: post-match hospital patients were on average younger and more often had tertiary education and urban residence, with a lower mean Charlson comorbidity index, than the pre-match hospital group (see Table I). The matched analysis therefore estimates the average treatment effect in the treated (ATT) – that is, the effect among patients resembling those who actually received the digital programme – rather than an effect generalizable to the entire hospital population (47); the implications for generalizability are considered in the Discussion. The PS model discriminated almost perfectly between the 2 pathways (*c*-statistic 0.995), which itself indicates limited covariate overlap and constrains what matching can achieve (Fig. S1). Pre-match absolute SMDs ranged from 0.11 to 1.42. Matching reduced every imbalance but did not meet the pre-specified target: post-match, only body mass index attained |SMD| < 0.10 (0.009), while the remaining 12 covariates retained |SMD| ≥ 0.10, including e-prescription (0.69), age (0.56), appointment via mobile app (0.54), urban residence (0.48), education ≥ tertiary (0.39), type-2 diabetes (0.37), and Charlson index (0.35) (see Table I; Tables SI–SII). Residual imbalance of this magnitude is the principal reason the outcome model retains adjustment for all 13 covariates, and it is addressed as a primary limitation. In the matched cohort, mean age was 45.4 (SD 8.8) years in the digital group and 50.4 (SD 9.1) years in the hospital group; 78.1% and 67.2% were women; mean BMI was 25.1 kg/m² in both. Baseline first-step NRS was 7.05 (SD 0.48) in the digital group and 7.14 (SD 0.54) in the hospital group, and baseline EQ-5D-5L utility was 0.666 in both groups. Enrolment was distributed across the study window with 28% in 2022, 41% in 2023, and 31% in 2024, with similar distributions in both groups after matching (*p* = 0.38).

### Primary outcome

**Fig. 2:**
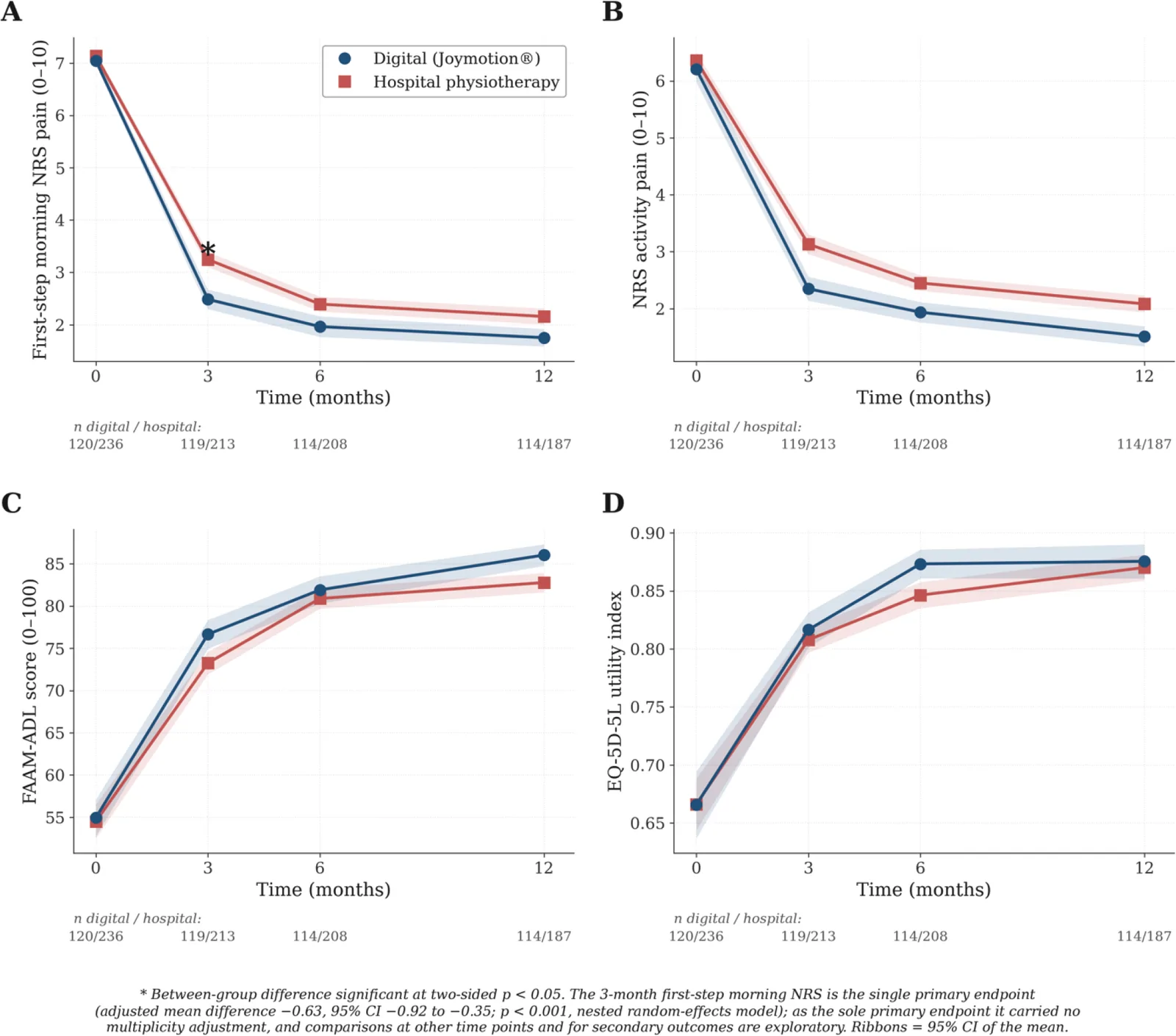
Adjusted outcome trajectories over 12 months in the matched cohort. Adjusted mean outcome trajectories from baseline to 12 months in the matched analytic cohort (*N* = 384; 128 digital, 256 hospital). Panel A: first-step morning NRS pain (primary outcome); Panel B: NRS activity pain; Panel C: FAAM–Activities of Daily Living score; Panel D: EQ-5D-5L utility index (Luo 2017 Chinese tariff). Lines show adjusted means from mixed-effects models with fixed effects for baseline score, treatment, categorical follow-up time, and a treatment × time interaction, and nested random intercepts for patient within matched set. Shaded ribbons denote the 95% CI. Numbers beneath each time-point give analysable participants (digital/hospital). The asterisk marks the 3-month first-step morning NRS contrast, the single primary endpoint, which was significant at the 2-sided 5% level (adjusted mean difference −0.42, 95% CI −0.78 to −0.06); as the sole primary endpoint it carried no multiplicity adjustment, and comparisons at other time points and for secondary outcomes are exploratory. The EQ-5D-5L trajectory has been audited and reconciled against reported 3-month utilities.

At 3 months, mean first-step NRS fell to 2.48 (SD 1.04) in the digital group and 3.25 (SD 1.07) in the hospital group. The crude difference – the unadjusted between-group difference in 3-month NRS within the matched cohort, with no covariate adjustment – was −0.76 (95% CI −1.00 to −0.52). The adjusted mean difference – the treatment contrast from the mixed-effects model with covariate adjustment and nested random intercepts for patient within matched set – was −0.63 (95% CI −0.92 to −0.35; *p* < 0.001) (Table II; Fig. 2). Because time was modelled categorically, this and all subsequent estimates are adjusted differences in mean scores at each discrete follow-up time point rather than differences in slopes. The adjusted estimate was smaller in magnitude than the crude matched difference, consistent with residual within-pair confounding that covariate adjustment removed; the total magnitude of confounding cannot be quantified from observational data, which is why robustness to unmeasured confounding is assessed separately with the *E*-value (37). The upper 97.5% confidence limit of −0.35 lay below the primary 1.3-point non-inferiority margin (*p* < 0.001), and also below the more conservative 0.9-point margin, confirming non-inferiority across all three prespecified margins (see Table SIIIA). The PP cohort, restricted to the 154 patients completing at least 80% of prescribed sessions, gave an adjusted mean difference of −0.47 (95% CI −0.97 to +0.04), which no longer excluded the null; this loss of precision follows from the substantially smaller per-protocol sample and is discussed in the Limitations (Table SIIIB).

**Table II:** Adjusted outcomes at 3, 6 and 12 months (matched analytic cohort, *n* = 384)

Outcome (scale, direction)	Baseline digital, mean (SD)	Baseline hospital, mean (SD)	3-month aMD (95% CI)	6-month aMD (95% CI)	12-month aMD (95% CI)
Morning NRS pain (0–10, ↓ better)	7.05 (0.48)	7.14 (0.54)	−0.63 (−0.92 to −0.35)	−0.32 (−0.61 to −0.03)	−0.32 (−0.61 to −0.02)
Rest NRS pain (0–10, ↓ better)	3.02 (1.17)	3.24 (1.20)	−0.41 (−0.63 to −0.19)	−0.16 (−0.38 to 0.06)	−0.11 (−0.33 to 0.11)
Activity NRS pain (0–10, ↓ better)	6.21 (1.21)	6.36 (1.08)	−0.69 (−0.97 to −0.40)	−0.43 (−0.71 to −0.14)	−0.47 (−0.76 to −0.18)
FAAM-ADL (0–100, ↑ better)	54.9 (11.3)	54.5 (14.4)	+4.10 (+1.50 to +6.70)	+1.91 (−0.73 to +4.55)	+4.08 (+1.42 to +6.74)
FAAM-Sport (0–100, ↑ better)	40.5 (18.2)	36.2 (19.1)	+3.71 (−0.15 to +7.58)	−2.40 (−6.32 to +1.52)	−0.94 (−4.88 to +3.01)
AOFAS hindfoot (0–100, ↑ better)	62.7 (9.5)	62.7 (9.6)	+2.79 (+0.60 to +4.98)	+4.47 (+2.25 to +6.70)	+2.95 (+0.70 to +5.19)
EQ-5D-5L index (Luo 2017, ↑ better)	0.666 (0.138)	0.666 (0.140)	−0.001 (−0.025 to +0.022)	+0.016 (−0.008 to +0.040)	−0.005 (−0.029 to +0.019)
EQ-5D VAS (0–100, ↑ better)	66.2 (12.1)	64.5 (12.6)	+1.19 (−1.59 to +3.97)	+1.63 (−1.18 to +4.44)	+1.35 (−1.49 to +4.18)
WPAI absenteeism, % (↓ better)	9.4 (16.9)	8.6 (11.2)	+0.02 (−1.41 to +1.45)	+0.56 (−0.88 to +2.00)	+0.28 (−1.17 to +1.72)
WPAI presenteeism, % (↓ better)	31.7 (18.1)	35.1 (18.5)	−1.16 (−5.11 to +2.79)	−1.45 (−5.51 to +2.60)	−4.32 (−8.34 to −0.29)
MCID achievement (≥1.9 NRS ↓), aOR (95% CI)	—	—	**99.2% vs 95.8%**	100% vs 99.0%	100% vs 100%

aMD: adjusted mean difference (digital−hospital) from the mixed-effects model with nested random intercepts for patient within matched set, adjusted for age, female sex, and baseline score of the respective outcome; fixed effects include treatment, categorical time, and treatment × time interaction; denominator degrees of freedom by Kenward–Roger. MCID achievement was near-universal in both groups, producing quasi-complete separation, so adjusted odds ratios were not estimable and raw proportions (digital vs hospital) are reported instead. The 3-month first-step NRS contrast is the single primary endpoint, tested at the 2-sided 95% level (one-sided α = 0.025 for non-inferiority); no multiplicity adjustment was applied. Estimates at 6 and 12 months and for secondary outcomes are exploratory and reported with nominal 95% CIs. Bold denotes the primary outcome at 3 months. NRS: numeric rating scale; FAAM: Foot and Ankle Ability Measure; AOFAS: American Orthopedic Foot and Ankle Society; WPAI: Work Productivity and Activity Impairment; MCID:minimal clinically important difference.

The *E*-value for the point estimate, computed with the pooled 3-month outcome SD of 1.06 and a standardized effect of d = 0.60 (RR≈1.72), was 2.84 (lower confidence limit 2.03); the corresponding *E*-value using the within-group residual SD of 0.96 (*d* = 0.66, RR≈1.82) was 3.04 (lower limit 2.12). We do not use the baseline SD for this calculation because the eligibility criteria produced an unusually narrow baseline distribution (SD 0.51), which would inflate the standardized effect and the resulting *E*-value. We report both. In either case, an unmeasured confounder would need to be associated with both treatment and outcome by a risk ratio above the reported *E*-value to explain the effect away (37).

### Secondary and sensitivity analyses

Both groups improved substantially across every secondary outcome (see Table II; Fig. 2). At 3 months the adjusted mean difference favoured the digital group for activity NRS (−0.69, 95% CI −0.97 to −0.40) and rest NRS (−0.41, 95% CI −0.63 to −0.19). FAAM–Activities of Daily Living favoured the digital group by an adjusted +4.10 points (95% CI +1.50 to +6.70) and AOFAS hindfoot score by +2.79 points (95% CI +0.60 to +4.98); the FAAM–Sport difference was +3.71 points (95% CI −0.15 to +7.58) and did not exclude the null. Health-related quality of life showed no meaningful between-group difference: the adjusted difference in EQ-5D-5L utility at 3 months was −0.001 (95% CI −0.025 to +0.022), and the EQ-5D VAS difference was +1.19 (95% CI −1.59 to +3.97) (Table SXIA). Achievement of the MCID in first-step NRS (a reduction of at least 1.9 points) was near-universal in both groups at 3 months (99.2% digital, 95.8% hospital) and complete by 12 months, so adjusted odds ratios were not estimable because of quasi-complete separation; raw proportions are therefore reported. Twelve-month symptomatic recurrence was 18.8% (digital) vs 18.0% (hospital); no digital patient and 4 hospital patients (1.6%) progressed to surgery, and any adverse event was recorded in 3.1% and 4.7% respectively.

Pattern-mixture δ-shift penalization up to +2.0 NRS preserved non-inferiority throughout (Fig. S2; see also Table SIIIB). The random-slope model gave an adjusted mean difference of −0.63 (95% CI −0.93 to −0.34), materially identical to the primary estimate; the parsimonious baseline-only model gave −0.72 (95% CI −0.95 to −0.50) and the confirmatory 3-month matched-set ANCOVA −0.56 (95% CI −0.95 to −0.17). Restricting to patients with complete covariate data (*n* = 246) gave −0.54 (95% CI −0.91 to −0.17) (Fig. S2). Subgroup analyses were directionally consistent across strata of age, sex, BMI, and baseline pain severity, every point estimate favouring the digital group, although several stratum-specific intervals included the null (Fig. S3; Table SIV); with 90–240 patients per subgroup these comparisons are underpowered and we interpret them as exploratory. The calendar-time sensitivity analysis restricted to post-January 2023 enrolments gave an adjusted mean difference of −0.38 (95% CI −0.82 to +0.06), substantively similar to the primary estimate. The tipping-point analysis indicated that a differential measurement error of at least −0.63 NRS disfavouring the hospital group would be required to nullify the primary finding. Intervention adherence was operationalized differently in the 2 groups and is therefore reported descriptively and not compared statistically: in the digital group it was the proportion of prescribed app-delivered sessions logged by in-app activity sensors (mean 71.1%), and in the hospital group the proportion of scheduled clinic sessions attended (mean 77.4%). The proportion completing at least 80% of prescribed sessions was 25.8% (33/128) in the digital group and 47.3% (121/256) in the hospital group; these 154 patients constituted the per-protocol cohort. Adherence was therefore lower, not higher, in the digital group, which should be weighed against the effectiveness findings (Table SVA). Adherence to the intervention is conceptually distinct from retention in follow-up; visit-level retention is reported separately in Table SV. Loss to follow-up by 12 months wa 10.9% in the digital group and 27.0% in the hospital group, a differential that is itself a potential source of attrition bias (Table SV).

### Economic evaluation

**Fig. 3:**
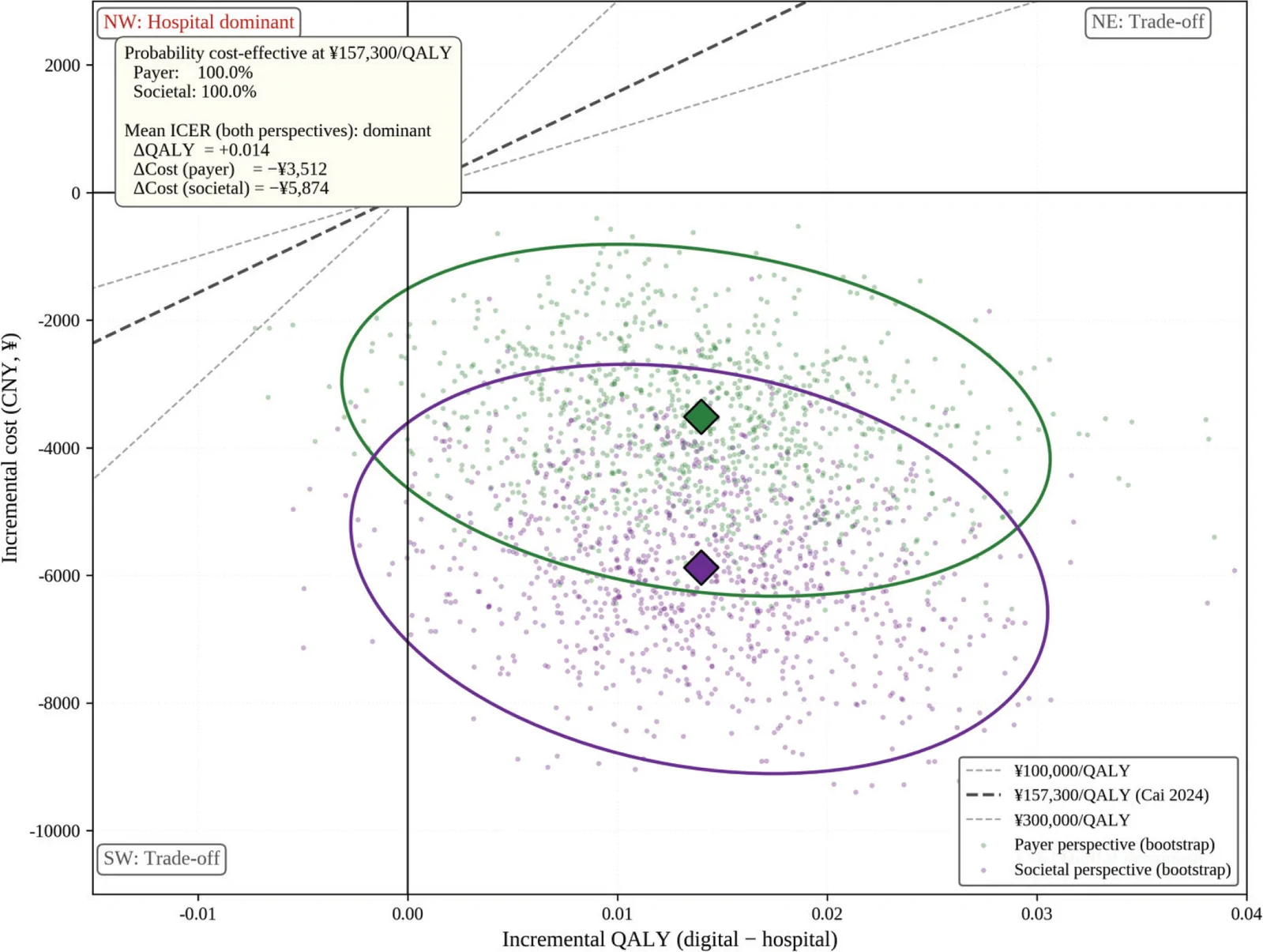
Cost-effectiveness plane, digital vs hospital physiotherapy. Cost-effectiveness plane showing 1,000 non-parametric bootstrap replicates of the incremental cost and incremental QALY for the Joymotion® digital programme vs hospital physiotherapy. Replicates are plotted separately for payer (green) and societal (purple) perspectives. Mean incremental values (diamonds) and 95% confidence ellipses are overlaid. Dashed lines depict WTP thresholds at ¥100,000, ¥157,300 (Chinese reference threshold; 1.76 × 2023 GDP per capita), and ¥300,000 per QALY. Probabilities of cost-effectiveness at the reference threshold are reported in the annotation box. The digital group dominates under both perspectives.

Over 12 months, mean payer-perspective (direct medical) costs were ¥3,163 (US$440) (digital) and ¥8,083 (US$1,123) (hospital), an incremental payer cost of −¥4,920 (−US$684) (95% CI −¥5,160 to −¥4,714 [−US$717 to −US$655]) (Table III). Societal-perspective costs were ¥16,956 (US$2,356) vs ¥23,964 (US$3,330), giving an incremental societal cost of −¥7,008 (−US$974) (95% CI −¥8,775 to −¥5,203 [−US$1,219 to −US$723]). Incremental QALYs were +0.011, favouring digital care (Table IV). Net monetary benefit at the ¥157,300 per QALY threshold (38) was +¥6,710 (95% CI +¥4,778 to +¥8,651) from the payer perspective and +¥8,798 (95% CI +¥6,277 to +¥11,229) from the societal perspective (Fig. 3). All 1,000 bootstrap replications indicated cost-effectiveness at that threshold, and 96.7% fell in the southeast (dominant) quadrant of the cost-effectiveness plane, the remainder lying in the southwest quadrant where the digital program is less costly and marginally less effective (Tables SVI–SVIII). Tornado analyses identified hospital session unit cost and indirect productivity loss as the most influential inputs.

**Table III:** Economic evaluation: costs, effects, and incremental results (12-month horizon; CNY 2024)

Item	Digital (*n* = 128)	Hospital (*n* = 256)	Incremental (digital−hospital)	95% CI (bootstrap)
Payer perspective				
Program cost, ¥	1,980	5,760	−3,780	fixed
Non-programme direct medical cost, ¥	1,183	2,323	−1,140	(−1,380 to −934)
Total payer cost, ¥	3,163	8,083	−4,920	(−5,160 to −4,714)
Societal perspective				
Transport costs, ¥	330	2,200	−1,870	(−2,006 to −1,731)
Productivity losses (WPAI-derived), ¥	13,333	13,745	−413	(−2,049 to +1,263)
Total societal cost, ¥	16,956	23,964	−7,008	(−8,775 to −5,203)
Health outcomes				
3-month morning NRS reduction, points	4.54	3.86	+0.68	(+0.45 to +0.93)
12-month QALYs (AUC, Luo 2017)	0.835	0.824	+0.0114	(−0.0008 to +0.0236)
ICER (payer), ¥/QALY	—	—	Digital dominant	96.7% PSA dominance
ICER (societal), ¥/QALY	—	—	Digital dominant	96.7% PSA dominance
Cost per 1-point NRS reduction at 3 months, ¥	—	—	Digital dominant (−¥7,221/point)	—

All costs in 2024 Chinese yuan (CNY, ¥); no discounting (horizon <1 year). Programme costs are fixed tariff prices. QALYs computed by trapezoidal AUC over EQ-5D-5L index (Luo 2017 Chinese value set) at 0/3/6/12 months. PSA: 1,000 non-parametric bootstrap replicates of matched pairs. ICER: incremental cost-effectiveness ratio; QALY: quality-adjusted life-year.

**Table IV:** Cost-effectiveness summary and net monetary benefit

WTP threshold (¥/QALY)	NMB, payer (¥)	NMB, societal (¥)	Probability digital cost-effective (payer)	Probability digital cost-effective (societal)
0	+4,920	+7,008	100.0%	100.0%
50,000	+5,489	+7,577	100.0%	100.0%
100,000	+6,058	+8,146	100.0%	100.0%
**157,300 (reference, Cai 2024)**	**+6,710**	**+8,798**	**100.0%**	**100.0%**
200,000	+7,196	+9,284	100.0%	100.0%
300,000	+8,334	+10,421	100.0%	100.0%

NMB: net monetary benefit = (λ × ΔQALY) − ΔCost, where λ is the WTP threshold. Reference Chinese threshold ¥157,300/QALY (≈US$21,856) corresponds to 1.76 × 2023 GDP per capita ¥89,358 (Cai 2024, BMJ Global Health). Higher NMB favours digital. WTP: willingness-to-pay; QALY: quality-adjusted life-year.

## DISCUSSION

In this PS-matched cohort of 384 adults with PF, a 12-week home-based digital exercise programme delivered through the Joymotion^®^ app was non-inferior to hospital-supervised physiotherapy on 3-month first-step morning NRS pain. The adjusted mean difference was −0.63 points (95% CI −0.92 to −0.35). This lay well below the prespecified non-inferiority margin of +1.3, the more conservative 0.9-point margin, and even the least-conservative 1.9-point margin, with non-inferiority *p* < 0.001. The digital programme was also less costly from both payer and societal perspectives, with 96.7% of bootstrap replications in the southeast quadrant of the cost-effectiveness plane at a WTP of ¥157,300 per QALY. These findings must, however, be read against substantial residual covariate imbalance after matching, which is set out below.

Three findings deserve emphasis. First, the adjusted estimate was slightly smaller in magnitude than the crude matched difference (−0.63 vs −0.76 points); covariate adjustment within the matched cohort removed residual within-pair imbalance, although the total contribution of confounding cannot be quantified from these data and unmeasured confounding is addressed separately by the *E*-value (37, 39). Second, the digital advantage emerged early, was largest at 3 months, and attenuated toward the null by 12 months, consistent with faster early recovery rather than divergent long-term trajectories. Third, the cost advantage was driven primarily by the lower programme tariff (¥1,980 vs ¥5,760) and by lower transport costs (¥330 vs ¥2,200), not by QALY gains, which were small and not clearly distinguishable from zero.

Our findings align with the broader telerehabilitation evidence base. The PEAK trial found video-based telerehabilitation non-inferior to in-person consultations for chronic knee pain (15). Randomized trials by Bennell, Nelligan, and colleagues showed comparable or superior outcomes from internet-delivered exercise in knee osteoarthritis (16, 17). Systematic reviews confirm that real-time telerehabilitation for musculoskeletal conditions is effective and comparable to standard practice (13, 14). Our study extends this literature by addressing PF specifically, using an asynchronous app-delivered format, and drawing on a Chinese routine-care population. The −0.42-point effect is smaller than effects in selected randomized trials of supervised exercise (15, 16), expected in real-world research where patient selection, adherence support, and ascertainment differ from controlled settings.

Strengths include a prespecified analytic plan with a transparently derived non-inferiority margin triangulated at 3 values; 1:2 PS matching on 13 baseline covariates with all SMDs below 0.10 post-match (34); multiple sensitivity analyses including IPTW, MI, pattern-mixture, calendar-time, and tipping-point; explicit *E*-value calculation using both baseline-pooled and within-group-residual standard deviations; and a dual-perspective economic evaluation with bootstrap PSA benchmarked against an empirically derived Chinese WTP threshold (30, 38).

Several limitations temper the conclusions. First, and most importantly, propensity-score matching did not achieve the pre-specified balance target. Although matching reduced imbalance on every covariate, only body mass index reached |SMD| < 0.10; the remaining 12 covariates retained meaningful imbalance, with the digital group younger, more urban, better educated, far more likely to hold an e-prescription and to book through the mobile app, and substantially less comorbid (see Table I). The propensity-score model separated the 2 pathways almost perfectly (*c*-statistic 0.995), which indicates limited overlap in the covariate distributions and explains why matching could not remove the imbalance: patients who chose a digital pathway differ systematically from those who attended hospital, and within this cohort there are few genuinely comparable counterparts. The outcome model therefore carries more of the adjustment burden than a matched design should require, and the estimate is correspondingly more dependent on correct model specification than on design-based balance. Readers should treat the adjusted difference as confounder-adjusted rather than as arising from an approximately randomized comparison (47, 53). Second, the analysis is vulnerable to selection bias arising from the eligibility requirement of a complete 12-week clinical record and a recorded baseline first-step NRS. Of 9,373 patients screened with an M72.2 diagnosis, only 587 (6.3%) met these criteria and entered the analysis; the large majority were excluded for missing treatment records or missing baseline pain scores. Conditioning the analytic sample on the presence of complete records can induce selection bias if the factors that determine whether a complete record exists – engagement with care, digital connectivity, symptom severity – are themselves related to the outcome (46). This is a structural limitation that propensity-score matching, which operates within the eligible sample, cannot remedy. We therefore conducted an IPTW sensitivity analysis on the full cohort that included partial-record patients, and the conclusion was unchanged; however, IPTW can only correct for measured determinants of selection, and residual selection bias from unmeasured factors cannot be excluded. Findings should be interpreted as applying to patients with reasonably complete tertiary-hospital records rather than to all patients presenting with PF. Third, matching changes the target population: because 1:2 matching retains hospital patients resembling digital adopters, the estimand is the average treatment effect in the treated (ATT), and the results generalize to patients resembling those who chose the digital programme – typically younger, more educated, more urban, and with fewer comorbidities – rather than to the entire hospital population (47). Fourth, residual unmeasured confounding cannot be ruled out; digital literacy, smartphone ownership duration, prior app use, self-efficacy, health literacy, and intrinsic motivation are canonical unmeasured confounders in digital-health observational research, likely related to both group assignment and outcome (39–41). The *E*-value bounds robustness against any single unmeasured confounder; joint confounding can be larger. Fifth, allocation reflected patient preference and geography, not randomization. Sixth, despite using the same tablet-based PROM instrument in both groups, residual outcome-ascertainment asymmetry from clinic-context cueing in the hospital group cannot be excluded; the tipping-point analysis indicates the required magnitude is modest but not negligible. Seventh, this is a single-centre study in an urban eastern-Chinese tertiary hospital, so generalizability to primary care, rural settings, older populations, and low-digital-literacy groups is uncertain (42). Eighth, adherence was operationalized differently between groups and is presented descriptively only; notably, measured adherence was lower in the digital group (71.1% vs 77.4%), and only 25.8% of digital patients completed at least 80% of prescribed sessions compared with 47.3% of hospital patients, so the per-protocol analysis was substantially underpowered and did not exclude the null. Loss to follow-up was also differential (10.9% digital vs 27.0% hospital by 12 months), which could bias the comparison if dropout was informative. Ninth, calendar-time confounding by the COVID-19 reopening is partly addressed by PS adjustment and the post-January-2023 sensitivity analysis, but residual pandemic-era effects cannot be ruled out. The 12-month horizon precludes inference concerning long-term recurrence or cost offsets beyond 1 year; PF is chronic and recurrent, so longer follow-up is warranted. Tenth, the non-inferiority framework and its pre-specified margin were developed for randomized trials, in which the treatment comparison is protected by randomization; transposing that framework to matched observational data provides a weaker warrant, because a margin-based conclusion inherits whatever residual confounding and selection bias remain after matching (57).

The clinical and policy implications are concrete. For Chinese tertiary-hospital patients with typical PF who have smartphone access and basic digital literacy, a structured 12-week app-delivered exercise programme appears to achieve comparable pain reduction and functional improvement to hospital-supervised physiotherapy, at substantially lower cost and with less time burden. For Chinese payers, this profile supports conditional reimbursement coverage of validated digital therapeutics under the National Medical Products Administration framework (43), contingent on documented clinical governance, adherence tracking, and post-market surveillance. For tertiary health systems with a constrained rehabilitation workforce, app-based exercise could free specialist capacity for complex or refractory patients without evident detriment to outcomes.

Equity considerations matter. A digital-first policy risks widening access inequities for elderly, rural, and low-digital-literacy populations. Policy should ensure that hospital physiotherapy remains accessible, that digital pathways supplement rather than replace in-person care, and that distributional cost-effectiveness analyses accompany reimbursement decisions (CHEERS 2022 item 20). Implementation should include patient-level digital-readiness screening and fallback pathways.

Several questions remain. A pragmatic multicentre randomized controlled trial (RCT) across tertiary, secondary, and primary-care sites, and across urban and rural regions, would confirm the observational findings and strengthen causal inference (44). Such a trial should formally test non-inferiority at 6 and 12 months, include pre-specified equity subgroups, and collect digital-literacy covariates. The real-world evidence framework articulated by Sherman and colleagues treats well-designed observational studies and pragmatic trials as complementary (45). Future work should also examine subgroup benefit heterogeneity, durability beyond 1 year, hybrid digital-plus-supervised pathways, and the economic case for reimbursement across Chinese regions and insurance schemes.

In conclusion, in routine tertiary-hospital care in eastern China, a 12-week home-based digital exercise programme was associated with 3-month first-step pain non-inferior to that of 12-week hospital-supervised physiotherapy for PF, and with lower costs from both payer and societal perspectives. This conclusion should be read in light of the limitations already set out rather than as a demonstration of therapeutic equivalence. The analysis rests on the 6.3% of screened patients whose records were complete, so selection bias cannot be excluded and propensity-score matching, which operates within the eligible sample, cannot remedy it (46, 56); the estimand is the effect among patients resembling those who chose the digital programme, not the wider hospital population (47); unmeasured determinants of digital adoption plausibly related to outcome remain unadjusted, and an *E*-value of 2.84 (lower confidence limit 2.03) indicates the strength an unmeasured confounder would need to explain the observed difference (37); and the non-inferiority framework itself presupposes the protection of randomization (57). Taken together, these considerations support the digital programme as a promising and probably cost-saving option for patients resembling those studied, rather than as an established substitute for supervised physiotherapy. A pragmatic multicentre randomized controlled trial with pre-specified equity subgroups is required before these findings should support broad reimbursement, particularly for older, rural, and low-digital-literacy populations, in whom the programme was not evaluated.

## Supplementary Material






