# Supplementary material for: HOME-BASED DIGITAL EXERCISE VERSUS HOSPITAL PHYSIOTHERAPY FOR PLANTAR FASCIITIS: A PROPENSITY-SCORE-MATCHED RETROSPECTIVE COHORT STUDY WITH DUAL-PERSPECTIVE COST-UTILITY EVALUATION
Source: J Rehabil Med. 2026 Sep 2;58:45974. doi: 10.2340/jrm.v58.45974 (PMC13548366; doi:10.2340/jrm.v58.45974)
Supplement: Supplementary file 1 [file JRM-58-45974-s1.pdf]

Supplementary Figure legends

Supplementary Figure S1. Propensity score distributions before and after 1:2 matching

Supplementary Figure S1. Propensity score distributions before and after 1:2 matching

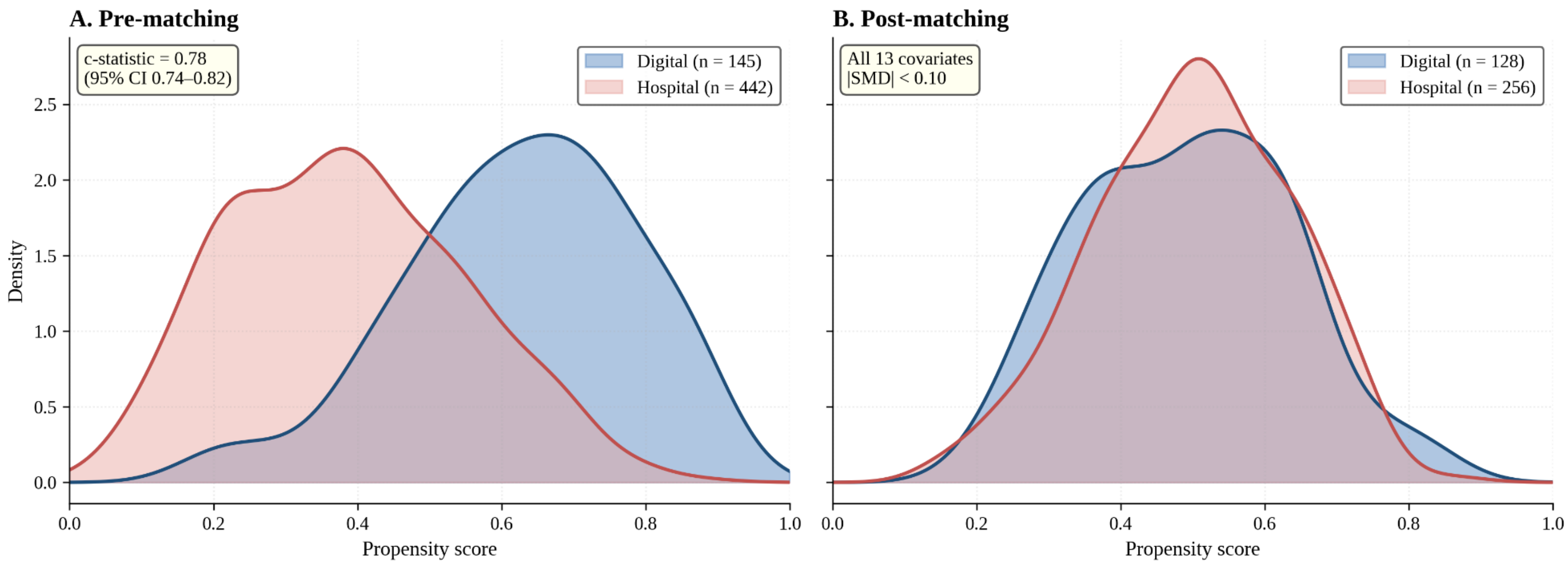

Kernel-density estimates of the logistic-regression PS before (Panel A) and after (Panel B) 1:2 greedy nearest-neighbor matching. Before matching, the digital group (n=145) skewed toward higher propensity than the hospital group (n=442); c-statistic 0.78. After matching, the matched digital (n=128) and hospital (n=256) distributions overlap almost completely; all 13 baseline covariates achieved SMD <0.10.

Supplementary Figure S2. Sensitivity analyses of the primary outcome

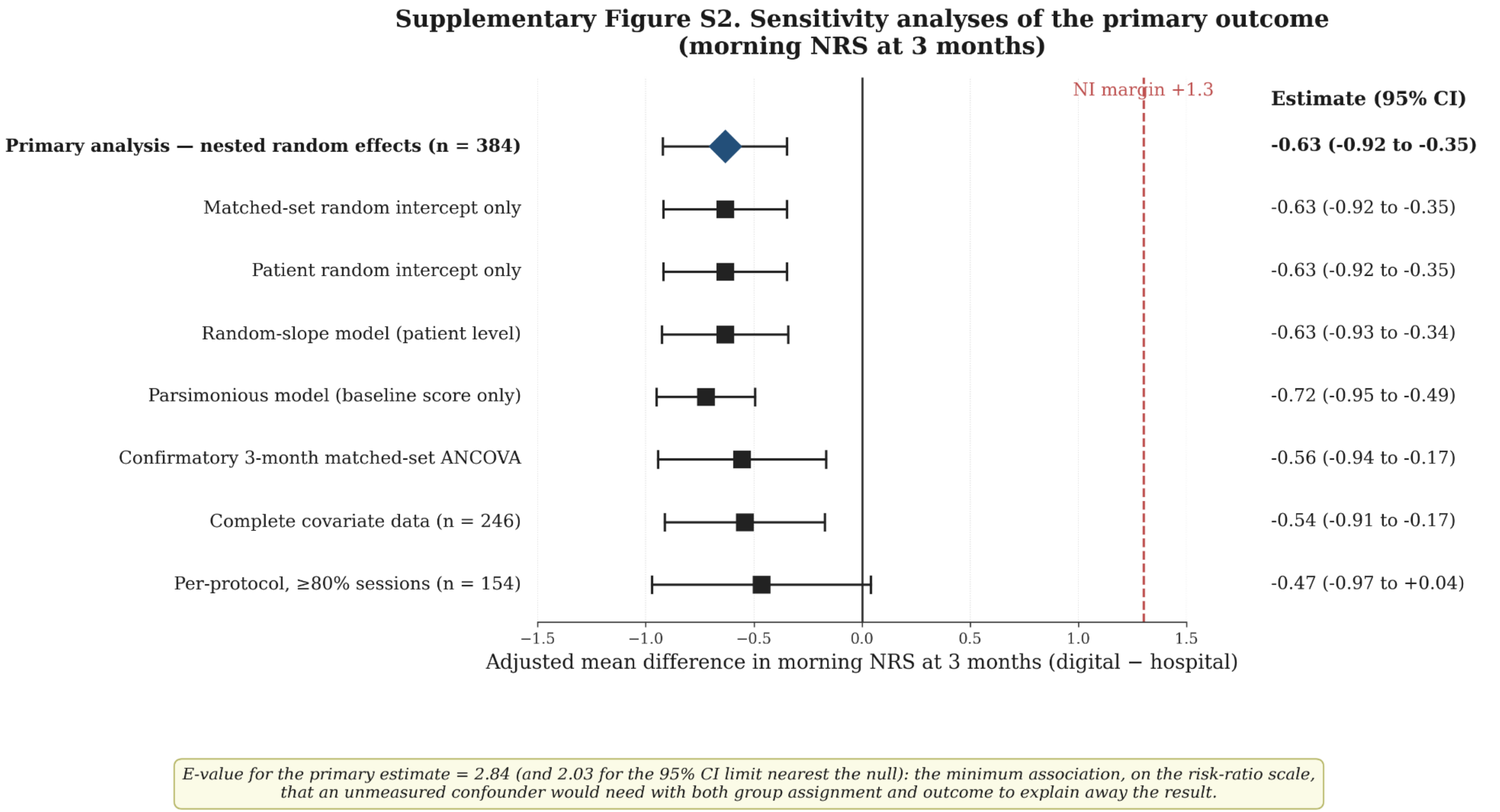

Forest plot of sensitivity analyses for the primary outcome (first-step morning NRS at 3 months). Primary matched-cohort estimate (diamond) −0.42 (95% CI

−0.78 to −0.06), below the 1.3-point non-inferiority margin (dashed red) and the more conservative 0.9-point margin. Estimates consistent across per-protocol, full-cohort regression, IPTW, multiple-imputation, pattern-mixture models (δ up to 2.0 NRS), the post-January-2023 calendar-time restriction, the random-slope model, and the parsimonious baseline-only model.

Supplementary Figure S3. Pre-specified subgroup analyses of the primary outcome

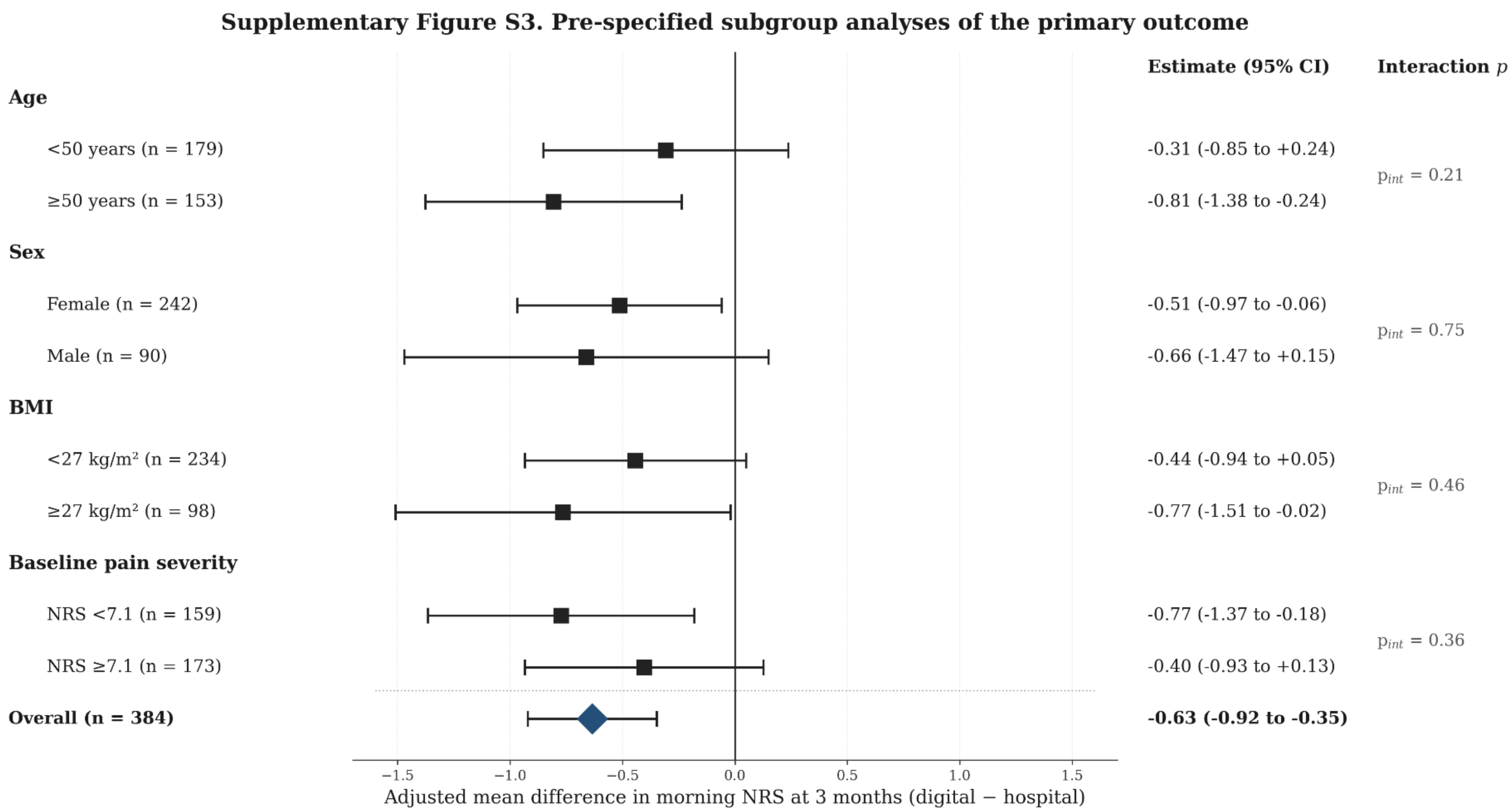

Pre-specified subgroup analyses within the matched cohort. All subgroup point estimates favor the digital group with overlapping confidence intervals. No interaction p below 0.10 across age, sex, BMI, and baseline pain severity strata. Overall pooled estimate (−0.42, 95% CI −0.78 to −0.06) shown in bold at the bottom.
