# Supplementary material for: HOME-BASED DIGITAL EXERCISE VERSUS HOSPITAL PHYSIOTHERAPY FOR PLANTAR FASCIITIS: A PROPENSITY-SCORE-MATCHED RETROSPECTIVE COHORT STUDY WITH DUAL-PERSPECTIVE COST-UTILITY EVALUATION
Source: J Rehabil Med. 2026 Sep 2;58:45974. doi: 10.2340/jrm.v58.45974 (PMC13548366; doi:10.2340/jrm.v58.45974)
Supplement: Supplementary file 2 [file JRM-58-45974-s2.pdf]

**Supplementary Table SI. Propensity-score model specification (multivariable logistic regression; outcome = digital program)**

| Covariate | β | SE | Odds ratio (95% CI) | p-value |
|---|---|---|---|---|
| Intercept | 0.62 | 0.84 | — | 0.46 |
| Age (per 10 years) | −0.38 | 0.09 | 0.68 (0.57–0.82) | <0.001 |
| Female (vs male) | −0.14 | 0.20 | 0.87 (0.59–1.29) | 0.49 |
| BMI (per 1 kg/m²) | −0.05 | 0.03 | 0.95 (0.90–1.01) | 0.09 |
| Education ≥ tertiary (vs < tertiary) | 0.46 | 0.21 | 1.58 (1.05–2.39) | 0.028 |
| Insurance URRBMI (vs UEBMI) | −0.29 | 0.24 | 0.75 (0.47–1.20) | 0.23 |
| Insurance out-of-pocket (vs UEBMI) | −0.08 | 0.36 | 0.92 (0.45–1.86) | 0.81 |
| Urban residence (vs rural) | 0.82 | 0.28 | 2.27 (1.31–3.93) | 0.003 |
| Appointment via app/WeChat (vs in-person) | 1.38 | 0.22 | 3.97 (2.58–6.11) | <0.001 |
| E-prescription flag | 0.71 | 0.21 | 2.03 (1.34–3.08) | <0.001 |
| Distance to hospital (per 10 km) | 0.22 | 0.06 | 1.24 (1.10–1.40) | <0.001 |
| Charlson index (per 1 unit) | −0.31 | 0.12 | 0.73 (0.58–0.93) | 0.010 |
| Baseline morning NRS (per 1 point) | −0.18 | 0.07 | 0.84 (0.73–0.96) | 0.012 |
| Prior PF episode | −0.26 | 0.22 | 0.77 (0.50–1.19) | 0.24 |
| Type-2 diabetes mellitus | −0.24 | 0.28 | 0.79 (0.45–1.37) | 0.39 |

Model diagnostics: c-statistic 0.78 (95% CI 0.74–0.82); Hosmer–Lemeshow $\chi^2 = 8.6$, df = 8, $p = 0.38$; Nagelkerke $R^2 = 0.29$; n = 587. UEBMI = Urban Employee Basic Medical Insurance; URRBMI = Urban-Rural Resident Basic Medical Insurance.

**Supplementary Table SII. Covariate balance pre- and post-matching (SMDs and variance ratios)**

| Covariate | Pre-match SMD | Post-match SMD | Pre-match variance ratio | Post-match variance ratio |
|---|---|---|---|---|
| Age | 0.55 | 0.04 | 0.80 | 0.95 |
| Female | 0.10 | 0.02 | — | — |
| BMI | 0.22 | 0.06 | 0.84 | 0.94 |
| Education ≥ tertiary | 0.35 | 0.03 | — | — |
| Urban residence | 0.42 | 0.03 | — | — |
| UEBMI | 0.24 | 0.01 | — | — |
| URRBMI | 0.22 | 0.01 | — | — |
| Out-of-pocket | 0.06 | 0.02 | — | — |
| Appointment via app/WeChat | 0.80 | 0.02 | — | — |
| E-prescription flag | 0.51 | 0.02 | — | — |
| Distance to hospital (log-km) | 0.46 | 0.05 | 1.18 | 1.06 |
| Charlson index | 0.43 | 0.05 | 0.72 | 0.83 |
| Baseline morning NRS | 0.32 | 0.06 | 0.88 | 0.94 |
| Prior PF episode | 0.20 | 0.03 | — | — |
| Type-2 diabetes mellitus | 0.23 | 0.04 | — | — |

All post-match |SMD| < 0.10 (target met); all post-match variance ratios within 0.8–1.25 (target met). Variance ratios not computed for binary covariates. SMD = standardized mean difference.

**Supplementary Table SIIIa. non-inferiority test of the primary outcome against three prespecified**

| Non-inferiority margin (NRS points) | Anchor derivation | Upper 97.5% CI of adjusted mean difference | Margin minus upper CI (margin) | p-value for non-inferiority | Conclusion |
|---|---|---|---|---|---|
| 0.9 (most conservative) | Landorf average-pain MID on 0–10 scale | −0.06 | +0.96 | <0.001 | Non-inferior |
| 1.3 (primary) | Midpoint of Landorf average-pain and first-step MIDs | −0.06 | +1.36 | <0.001 | Non-inferior |
| 1.9 (least conservative) | Landorf first-step MID on 0–10 scale | −0.06 | +1.96 | <0.001 | Non-inferior |

Non-inferiority test of the primary outcome (first-step morning NRS at 3 months) against three prespecified margins. The adjusted mean difference was −0.42 (95% CI −0.78 to −0.06). Non-inferiority is declared if the upper 97.5% confidence limit of −0.06 lies below the stated margin. All three margins yield the same non-inferiority conclusion, with p<0.001 under the most conservative 0.9-point margin. This table was previously presented as main manuscript Table 3 and has been relocated to the supplementary materials; its content is unchanged.

**Supplementary Table SIIIb. Per-protocol sensitivity analysis (cohort completing at least 80% of prescribed sessions)**

| Outcome at 3 months | PP n | PP aMD (95% CI) | Matched-cohort aMD (95% CI) | Δ (PP − matched cohort) |
|---|---|---|---|---|
| Morning NRS pain | 291 | −0.48 (−0.86 to −0.10) | −0.42 (−0.78 to −0.06) | −0.06 |
| Activity NRS pain | 291 | −0.44 (−0.80 to −0.08) | −0.38 (−0.73 to −0.03) | −0.06 |
| FAAM-ADL | 291 | +3.2 (+0.6 to +5.8) | +2.8 (+0.3 to +5.3) | +0.4 |
| EQ-5D-5L index | 291 | +0.024 (+0.006 to +0.042) | +0.021 (+0.004 to +0.038) | +0.003 |

Per-protocol (PP) cohort restricted to the 291 patients (75.8% of the matched analytic cohort) completing at least 80% of prescribed sessions. aMD = adjusted mean difference (digital − hospital) from mixed-effects model.

**Supplementary Table SIV. Subgroup analyses for the primary outcome (3-month morning NRS; matched analytic cohort)**

| Subgroup | n (digital / hospital) | aMD (95% CI) | Interaction p |
|---|---|---|---|
| Age < 50 years | 68 / 136 | −0.45 (−0.92 to +0.02) | 0.84 |
| Age ≥ 50 years | 60 / 120 | −0.39 (−0.90 to +0.12) | |
| Female | 82 / 167 | −0.44 (−0.88 to 0.00) | 0.79 |
| Male | 46 / 89 | −0.38 (−1.00 to +0.24) | |
| BMI < 27 | 75 / 148 | −0.40 (−0.86 to +0.06) | 0.91 |
| BMI ≥ 27 | 53 / 108 | −0.44 (−1.00 to +0.12) | |
| Baseline NRS < 7 | 66 / 131 | −0.32 (−0.79 to +0.15) | 0.42 |
| Baseline NRS ≥ 7 | 62 / 125 | −0.53 (−1.04 to −0.02) | |

Effects are broadly homogeneous across subgroups; no interaction $p < 0.10$. aMD = adjusted mean difference (digital − hospital) from the mixed-effects model with nested random intercepts for patient within matched set, stratum × treatment interaction term, and covariate adjustment for age, sex and baseline score.

**Supplementary Table SV. Visit-level dropout rates by treatment group and time point**

| Visit | Digital completed (n/128, %) | Hospital completed (n/256, %) | Dropout digital (%) | Dropout hospital (%) | $\chi^2$ p-value |
|---|---|---|---|---|---|
| Baseline | 128 (100.0) | 256 (100.0) | 0.0 | 0.0 | — |
| 3 months | 122 (95.3) | 239 (93.4) | 4.7 | 6.6 | 0.46 |
| 6 months | 115 (89.8) | 220 (85.9) | 10.2 | 14.1 | 0.28 |
| 12 months | 109 (85.2) | 209 (81.6) | 14.8 | 18.4 | 0.39 |

Missingness is visit-level holistic (all instruments missing if a visit was not attended); no item-level imputation was performed. Mixed-effects maximum-likelihood estimation handles the missing-at-random (MAR) assumption. Little's MCAR test was not applicable given the visit-level holistic missingness structure.

**Supplementary Table SVa. Intervention adherence (descriptive)**

| Adherence metric | Digital (n=128) | Hospital (n=256) | Definition / note |
|---|---|---|---|
| Operational definition of a completed session | Prescribed app session logged by in-app activity sensors | Scheduled clinic session attended | Definitions differ by delivery mode and are not directly comparable |
| Mean session completion, % | 71.1 | 77.4 | Descriptive only; not compared statistically |
| Patients completing ≥80% of prescribed sessions, n (%) | 33 (25.8) | 121 (47.3) | These 154 patients constitute the per-protocol cohort (Table S3b) |
| Relationship to follow-up retention | Distinct from visit retention | Distinct from visit retention | Visit-level retention is reported separately in Table S5 |

Adherence to the prescribed intervention was operationalized differently in the two groups (app-sensor session logs in the digital group; clinic attendance in the hospital group) and is therefore presented descriptively and not compared statistically. Adherence is conceptually distinct from retention in follow-up (Supplementary Table S5). The count of patients completing at least 80% of prescribed sessions (33 + 121 = 154) defines the per-protocol cohort analyzed in Supplementary Table S3b.

## Supplementary Table SVb. Variance components and intraclass correlations from the nested random-effects primary model

| Variance component | Variance estimate | SD | Interpretation |
|---|---|---|---|
| Matched set, $u_k$ | 0.000 | 0.000 | Between-matched-set variation induced by 1:2 propensity-score matching |
| Patient within matched set, $v_{=k}$ | 0.0001 | 0.011 | Between-patient variation; the source of within-patient correlation across the 3-, 6- and 12-month measurements |
| Residual, $\varepsilon_{a=k}$ | 0.929 | 0.964 | Observation-level (within-patient) variation |
| ICC, matched set | 0.000 | — | $\sigma^2(u) / [\sigma^2(u) + \sigma^2(v) + \sigma^2(\varepsilon)]$: correlation between two measurements on different patients in the same matched set |
| ICC, within patient | 0.0001 | — | $[\sigma^2(u) + \sigma^2(v)] / [\sigma^2(u) + \sigma^2(v) + \sigma^2(\varepsilon)]$: correlation between two measurements on the same patient |

Variance components from the primary mixed-effects model with nested random intercepts for patient within matched set (Supplementary S5). Reporting both components allows readers to verify that the two sources of clustering, matching-induced and repeated-measures, are separately identified, and to judge the magnitude of the within-patient correlation that a matched-set-only random intercept would have misrepresented. Values are means across the 20 imputed datasets from the primary model. Both clustering variances are negligible after adjustment for baseline score and the 13 covariates, so the intraclass correlations are effectively zero and the three candidate random-effects structures give identical estimates. Before covariate adjustment the patient-level variance is 0.151 against a residual of 0.940, an unadjusted within-patient intraclass correlation of 0.138, indicating that the repeated-measures correlation is real but is fully explained by the baseline score and covariates.

**Supplementary Table SVI. Cost component breakdown (mean per patient over 12 months, ¥ 2024)**

| Cost category | Digital (n=128) | Hospital (n=256) | Difference (digital − hospital) |
|---|---|---|---|
| **Payer perspective** | | | |
| Program tariff | 1,980 | 5,760 | −3,780 |
| Physician consultations | 486 | 412 | +74 |
| Imaging (X-ray / ultrasound / MRI) | 624 | 512 | +112 |
| Medications (NSAIDs, injections, TCM topical) | 812 | 728 | +84 |
| Injections/procedures (incl. ESWT) | 316 | 318 | −2 |
| **Subtotal, payer** | **4,218** | **7,730** | **−3,512** |
| **Societal add-ons** | | | |
| Transport (patient + caregiver) | 412 | 1,486 | −1,074 |
| Absenteeism (hourly wage × WPAI days) | 648 | 1,284 | −636 |
| Presenteeism (productivity loss) | 1,124 | 1,776 | −652 |
| **Subtotal, societal add-ons** | **2,184** | **4,546** | **−2,362** |
| **Total, societal perspective** | **6,402** | **12,276** | **−5,874** |

Transport costs are higher in the hospital group because of approximately 24 return trips over 12 weeks versus approximately 2 hospital visits in the digital group. Productivity losses valued using the human-capital approach with 2024 Chinese urban average hourly wage of ¥41.6. TCM = traditional Chinese medicine; ESWT = extracorporeal shock wave therapy; NSAID = non-steroidal anti-inflammatory drug.

**Supplementary Table SVII. Probabilistic sensitivity analysis (1,000 bootstrap iterations)**

| Cost-effectiveness plane quadrant | Payer (%) | Societal (%) | Interpretation |
|---|---|---|---|
| South-east (less costly, more effective) | 96.8 | 99.1 | Digital dominant |
| North-east (more costly, more effective) | 2.1 | 0.6 | Trade-off, requires ICER assessment |
| South-west (less costly, less effective) | 1.0 | 0.3 | Trade-off, requires ICER assessment |
| North-west (more costly, less effective) | 0.1 | 0.0 | Hospital dominant |

Bootstrap resampling performed at the patient-pair level within the matched cohort to preserve the 1:2 matching structure. Percentages may not sum to 100 because of rounding. ICER = incremental cost-effectiveness ratio.

**Supplementary Table SVIII. Deterministic (tornado) sensitivity analysis on incremental NMB at ¥157,300/QALY (payer perspective)**

| Parameter varied ±20% | Low NMB (¥) | Base NMB (¥) | High NMB (¥) | Range | Conclusion stable? |
|---|---|---|---|---|---|
| Digital program tariff (¥1,980) | 5,318 | 5,714 | 6,110 | 792 | Dominant |
| Hospital program tariff (¥5,760) | 4,562 | 5,714 | 6,866 | 2,304 | Dominant |
| Treatment effect on QALY (+0.014) | 5,274 | 5,714 | 6,154 | 880 | Dominant |
| Digital adherence (71.1%) | 5,162 | 5,714 | 6,266 | 1,104 | Dominant |
| Reimbursement ratio (65%) | 5,402 | 5,714 | 6,026 | 624 | Dominant |
| Non-program direct medical cost | 5,446 | 5,714 | 5,982 | 536 | Dominant |
| Transport cost (societal) | 5,500 | 5,714 | 5,928 | 428 | Dominant |
| Productivity losses (societal) | 5,456 | 5,714 | 5,972 | 516 | Dominant |

Hospital program tariff is the single most influential parameter; digital dominance is preserved under all ±20% perturbations. NMB = net monetary benefit; QALY = quality-adjusted life-year.

**Supplementary Table SIX. Extraction codes and terminology used to identify plantar fasciitis, comorbidities, procedures, laboratory investigations, imaging, medications and rehabilitation interventions in the Chinese tertiary hospital EHR / HIS**

| Category | Code system | Code | Chinese name | English name | Notes / Use in study |
|---|---|---|---|---|---|
| **1. Diagnosis codes (ICD-10, Chinese Clinical Modification – GB/T 14396-2016)** | | | | | |
| Diagnosis | ICD-10-CM (China) | M72.2 | 足底筋膜炎 / 足底筋膜纤维瘤病 | Plantar fasciitis / plantar fascial fibromatoses | Primary case-defining diagnosis for cohort entry |
| Diagnosis | ICD-10-CM (China) | M72.200 | 足底筋膜炎 | Plantar fasciitis (6-digit Chinese extension) | Alternative granular code accepted in HIS |
| Diagnosis | ICD-10-CM (China) | M77.3 | 跟骨骨刺 | Calcaneal (heel) spur | Frequently co-coded with M72.2 |
| Diagnosis | ICD-10-CM (China) | M76.6 | 跟腱炎 | Achilles tendinitis | Differential / co-existing enthesopathy |
| Diagnosis | ICD-10-CM (China) | M76.8 | 下肢其他特指的肌腱端病 (胫后肌腱炎) | Posterior tibial tendinitis (other specified enthesopathies of lower limb) | Captures posterior tibial tendinopathy |
| Diagnosis | ICD-10-CM (China) | M21.4 | 扁平足 (获得性) | Pes planus / acquired flat foot | Biomechanical risk factor |

| Category | Code system | Code | Chinese name | English name | Notes / Use in study |
|---|---|---|---|---|---|
| Diagnosis | ICD-10-CM (China) | M20.1 | 拇外翻 (后天性) | Hallux valgus (acquired) | Biomechanical risk factor |
| Diagnosis | ICD-10-CM (China) | G57.5 | 跗管综合征 | Tarsal tunnel syndrome | Differential diagnosis of medial heel pain |
| Diagnosis | ICD-10-CM (China) | M77.4 | 跖骨痛 | Metatarsalgia | Differential / forefoot comparator |
| Diagnosis | ICD-10-CM (China) | M79.67 | 足和趾痛 | Pain in foot and toes (unspecified) | Non-specific heel/foot pain |
| Diagnosis | ICD-10-CM (China) | M77.9 | 未特指的肌腱端病 | Enthesopathy, unspecified (of lower limb) | Captures non-specific entheseal disorders |
| Diagnosis | ICD-10-CM (China) | M25.57 | 足和踝关节痛 | Pain in foot and ankle joint | Secondary pain code |
| Diagnosis | ICD-10-CM (China) | S93.4 | 踝关节韧带扭伤和劳损 | Sprain/strain of ligaments of ankle | Prior injury history |
| Diagnosis | ICD-10-CM (China) | M19.07 | 踝和足原发性骨关节病 | Primary osteoarthritis, ankle and foot | Co-morbid OA of foot/ankle |
| Diagnosis | ICD-10-CM (China) | E11.9 | 2型糖尿病，不伴有并发症 | Type 2 diabetes mellitus, without complications | Comorbidity (metabolic) |
| Diagnosis | ICD-10-CM (China) | E66.9 | 肥胖，未特指 | Obesity, unspecified | Key risk factor / adjustment covariate |

| Category | Code system | Code | Chinese name | English name | Notes / Use in study |
|---|---|---|---|---|---|
| Diagnosis | ICD-10-CM (China) | I10 | 特发性 (原发性) 高血压 | Essential (primary) hypertension | Cardiovascular comorbidity |
| Diagnosis | ICD-10-CM (China) | M10.9 | 痛风，未特指 | Gout, unspecified | Differential (metatarsophalangeal pain) |
| Diagnosis | ICD-10-CM (China) | M06.9 | 类风湿关节炎，未特指 | Rheumatoid arthritis, unspecified | Inflammatory differential |
| Diagnosis | ICD-10-CM (China) | M45 | 强直性脊柱炎 | Ankylosing spondylitis | Seronegative spondyloarthropathy differential |
| **2. Procedure codes (ICD-9-CM-3, Chinese National Clinical Version 2.0)** | | | | | |
| Procedure | ICD-9-CM-3 | 83.14 | 筋膜切开术 (足底筋膜切断/松解术) | Fasciotomy – plantar fascia release | Surgical release of plantar fascia |
| Procedure | ICD-9-CM-3 | 83.14x001 | 足底筋膜切断术 | Plantar fasciotomy (Chinese extension code) | 6-digit granular extension used in HIS |
| Procedure | ICD-9-CM-3 | 83.39 | 其他软组织病损切除术 | Excision of other soft tissue lesion | Open plantar fascia debridement where applicable |

| Category | Code system | Code | Chinese name | English name | Notes / Use in study |
|---|---|---|---|---|---|
| Procedure | ICD-9-CM-3 | 83.21 | 软组织活组织检查 | Biopsy of soft tissue | Rarely used – nodular lesions |
| Procedure | ICD-9-CM-3 | 99.23 | 类固醇注射 | Injection of steroid | Local corticosteroid injection |
| Procedure | ICD-9-CM-3 | 99.29 | 其他治疗性物质的注射或输注 | Injection/infusion of other therapeutic substance | Used for PRP / local analgesic blocks |
| Procedure | ICD-9-CM-3 | 93.39 | 其他物理治疗 (含体外冲击波治疗) | Other physical therapy (incl. ESWT) | Principal code for ESWT sessions in HIS |
| Procedure | ICD-9-CM-3 | 93.39x005 | 体外冲击波治疗 | Extracorporeal shock wave therapy (ESWT) | 6-digit Chinese extension for ESWT |
| Procedure | ICD-9-CM-3 | 93.35 | 其他热疗法 (蜡疗、红外线) | Other heat therapy (paraffin, infrared) | Captures paraffin wax therapy |
| Procedure | ICD-9-CM-3 | 93.34 | 超声透热疗法 | Diathermy by ultrasound | Therapeutic ultrasound |
| Procedure | ICD-9-CM-3 | 93.36 | 短波透热疗法 | Shortwave diathermy | Shortwave electrotherapy |
| Procedure | ICD-9-CM-3 | 93.38 | 综合物理治疗 | Combined physical therapy | Medium-frequency / interferential electrotherapy |
| Procedure | ICD-9-CM-3 | 00.01 | 诊断性超声引导 (操作性超声) | Ultrasound guidance for procedures | Ultrasound-guided injection |

| Category | Code system | Code | Chinese name | English name | Notes / Use in study |
|---|---|---|---|---|---|
| Procedure | ICD-9-CM-3 | 93.27 | 伸展/拉伸训练 | Stretching of muscle / tendon | Supervised plantar fascia / gastrocnemius stretching |
| Procedure | ICD-9-CM-3 | 93.11 | 辅助性运动训练 | Assisted exercise | Functional exercise instruction |
| Procedure | ICD-9-CM-3 | 93.23 | 矫形鞋装配 (足弓支撑) | Fitting of orthotic shoe / arch support | Custom insole / orthotic fitting |
| Procedure | ICD-9-CM-3 | 93.54 | 夹板 (夜间夹板) 应用 | Application of splint (night splint) | Night splint for PF |
| Procedure | ICD-9-CM-3 | 99.91 / 99.92 | 针灸 / 电针 | Acupuncture / electroacupuncture (TCM) | Captured under TCM module of HIS |
| Procedure | ICD-9-CM-3 | local | 推拿治疗 | Tuina (Chinese therapeutic massage) | Captured via TCM HIS module |
| Procedure | ICD-9-CM-3 | local | 中药熏洗 | TCM herbal fumigation / soaking | Local Chinese extension code |
| **3. Laboratory tests (Chinese lab dictionary; LOINC-compatible)** | | | | | |
| Laboratory | Local / LOINC | 58410-2 | 血常规 (全血细胞计数) | Complete blood count (CBC) | Baseline workup |

| Category | Code system | Code | Chinese name | English name | Notes / Use in study |
|---|---|---|---|---|---|
| Laboratory | Local / LOINC | 30341-2 | 红细胞沉降率 (血沉, ESR) | Erythrocyte sedimentation rate | Inflammatory marker |
| Laboratory | Local / LOINC | 1988-5 | 超敏C反应蛋白 / C反应蛋白 | C-reactive protein (CRP / hs-CRP) | Inflammatory marker |
| Laboratory | Local / LOINC | 4548-4 | 糖化血红蛋白 (HbA1c) | Glycated hemoglobin | Diabetes comorbidity / glycaemic control |
| Laboratory | Local / LOINC | 1558-6 | 空腹血糖 (FPG) | Fasting plasma glucose | Diabetes screening |
| Laboratory | Local / LOINC | 3084-1 | 血尿酸 | Serum uric acid | Exclude gout |
| Laboratory | Local / LOINC | 11572-5 | 类风湿因子 (RF) | Rheumatoid factor | Exclude RA |
| Laboratory | Local / LOINC | 32035-8 | 抗环瓜氨酸肽抗体 | Anti-CCP antibody | Exclude RA |
| Laboratory | Local / LOINC | 4842-1 | 人类白细胞抗原B27 | HLA-B27 | Exclude spondyloarthropathy |
| Laboratory | Local / LOINC | 62292-8 | 25-羟基维生素D | 25-hydroxyvitamin D | Musculoskeletal pain risk factor |
| Laboratory | Local HIS | 39156-5 | 体重指数 (BMI) 测定 | Body-mass index measurement | Anthropometric covariate |
| **4. Medications (Chinese generic and trade names – ATC codes NOT** | | | | | |

| Category | Code system | Code | Chinese name | English name | Notes / Use in study |
|---|---|---|---|---|---|
| **used in Chinese EHRs)** | | | | | |
| NSAID (oral) | Chinese generic/trade | — | 塞来昔布胶囊 (西乐葆) | Celecoxib capsules (Celebrex®) | COX-2 selective NSAID |
| NSAID (oral) | Chinese generic/trade | — | 双氯芬酸钠缓释片 (英太青) | Diclofenac sodium sustained-release tablets | Non-selective NSAID |
| NSAID (oral) | Chinese generic/trade | — | 布洛芬缓释胶囊 (芬必得) | Ibuprofen sustained-release capsules | Non-selective NSAID |
| NSAID (oral) | Chinese generic/trade | — | 洛索洛芬钠片 (乐松) | Loxoprofen sodium tablets | Pro-drug NSAID, widely used in China |
| NSAID (oral) | Chinese generic/trade | — | 美洛昔康片 (莫比可) | Meloxicam tablets | Preferential COX-2 NSAID |
| NSAID (oral) | Chinese generic/trade | — | 依托考昔片 (安康信) | Etoricoxib tablets | COX-2 selective NSAID |
| Non-opioid analgesic | Chinese generic/trade | — | 对乙酰氨基酚片 | Paracetamol tablets | First-line analgesic |
| Compound analgesic | Chinese generic/trade | — | 氨酚待因片 | Paracetamol/codeine compound tablets | Moderate pain |
| Weak opioid | Chinese generic/trade | — | 盐酸曲马多缓释片 | Tramadol SR tablets | Refractory pain |
| Corticosteroid injection | Chinese generic/trade | — | 醋酸曲安奈德注射液 (肯纳可) | Triamcinolone acetonide injection | Local corticosteroid injection |

| Category | Code system | Code | Chinese name | English name | Notes / Use in study |
| --- | --- | --- | --- | --- | --- |
| Corticosteroid injection | Chinese generic/trade | — | 复方倍他米松注射液 (得宝松) | Compound betamethasone injection (Diprospan®) | Long-acting injection |
| Local anaesthetic | Chinese generic/trade | — | 盐酸利多卡因注射液 | Lidocaine injection | Adjunct for injection procedures |
| NSAID (topical) | Chinese generic/trade | — | 双氯芬酸二乙胺乳胶剂 (扶他林) | Diclofenac diethylamine emulgel (Voltaren®) | Topical NSAID |
| NSAID (topical patch) | Chinese generic/trade | — | 氟比洛芬凝胶贴膏 (泽普思) | Flurbiprofen gel patch | Topical NSAID patch |
| TCM external | Chinese generic/trade | — | 云南白药膏 | Yunnan Baiyao medicated plaster | Proprietary TCM topical analgesic |
| TCM external | Chinese generic/trade | — | 活血止痛膏 | Huoxue Zhitong medicated plaster | TCM analgesic plaster |
| TCM oral | Chinese generic/trade | — | 骨刺平片 | Gucipin tablets | Proprietary TCM for heel spur / PF |
| TCM oral | Chinese generic/trade | — | 独一味胶囊 | Duyiwei capsules | TCM analgesic / haemostatic |
| Neurotrophic | Chinese generic/trade | — | 甲钴胺片 (弥可保) | Mecobalamin tablets | Neuropathic pain component |
| **5. Imaging procedures (RIS dictionary; ICD-9-** | | | | | |

| Category | Code system | Code | Chinese name | English name | Notes / Use in study |
|---|---|---|---|---|---|
| **CM-3 where applicable)** | | | | | |
| Imaging | ICD-9-CM-3 / RIS | 88.28 | 足踝X线片 (正侧位) | Foot / ankle plain radiograph (AP + lateral) | Rule out spur, fracture, arthritis |
| Imaging | ICD-9-CM-3 / RIS | 88.28x00x | 负重位足侧位X线片 | Weight-bearing lateral foot radiograph | Arch height / calcaneal pitch |
| Imaging | ICD-9-CM-3 / RIS | 88.79 | 足底筋膜超声 (肌骨超声) | Musculoskeletal ultrasound of plantar fascia | Fascia thickness (>4 mm), hypoechogenicity |
| Imaging | ICD-9-CM-3 / RIS | 88.94 | 足踝MRI | Magnetic resonance imaging of foot / ankle | Confirm PF, exclude stress fracture |
| Imaging | ICD-9-CM-3 / RIS | 92.14 | 骨扫描 / 骨显像 | Bone scan / bone scintigraphy | Exclude stress fracture, inflammatory arthropathy |
| **6. Rehabilitation and TCM procedures (HIS rehabilitation / TCM modules)** | | | | | |
| Rehabilitation | ICD-9-CM-3 / HIS | 93.39x005 | 体外冲击波治疗 (ESWT) | Extracorporeal shock wave therapy | Focused / radial ESWT, 3–5 sessions typical |

| Category | Code system | Code | Chinese name | English name | Notes / Use in study |
|---|---|---|---|---|---|
| Rehabilitation – TCM | HIS TCM module | 99.91 | 针灸 (体针) | Acupuncture (manual) | TCM intervention |
| Rehabilitation – TCM | HIS TCM module | 99.92 | 电针 | Electroacupuncture | TCM + electrical stimulation |
| Rehabilitation – TCM | HIS TCM module | local | 推拿 (手法治疗) | Tuina | TCM manual therapy |
| Rehabilitation | ICD-9-CM-3 | 93.39 | 理疗 (综合物理治疗) | Physiotherapy, general | Umbrella code |
| Rehabilitation | ICD-9-CM-3 | 93.34 | 超声波治疗 | Therapeutic ultrasound | Continuous / pulsed 1 MHz |
| Rehabilitation | ICD-9-CM-3 | 93.36 | 短波治疗 | Shortwave diathermy | Deep-heating modality |
| Rehabilitation | ICD-9-CM-3 | 93.38 | 中频电疗 | Medium-frequency electrotherapy | Interferential / TENS-type |
| Rehabilitation | ICD-9-CM-3 | 93.35 | 蜡疗 | Paraffin wax therapy | Superficial heat |
| Rehabilitation – TCM | HIS TCM module | local | 中药熏洗 (足浴) | TCM herbal fumigation / foot-soak | TCM external therapy |
| Rehabilitation | ICD-9-CM-3 | 93.27 | 拉伸训练 (足底筋膜 / 小腿三头肌) | Stretching (plantar fascia / triceps surae) | Supervised stretching |
| Rehabilitation | ICD-9-CM-3 | 93.23 | 足弓支撑 / 矫形鞋垫装配 | Arch support / custom insole fitting | Orthotic prescription |
| Rehabilitation | ICD-9-CM-3 | 93.54 | 夜间夹板 (背伸夹板) | Night splint (dorsiflexion) | Overnight splinting |

| Category | Code system | Code | Chinese name | English name | Notes / Use in study |
|---|---|---|---|---|---|
| Rehabilitation | ICD-9-CM-3 | 93.11 | 功能锻炼指导 | Functional exercise instruction | Therapist-supervised home program |

ICD-10 diagnosis codes follow the Chinese Clinical Modification (ICD-10-CM China, National Clinical Version 2.0 / GB/T 14396-2016). Procedures use ICD-9-CM-3 (Chinese National Clinical Version 2.0) as mandated by the NHSA for inpatient records and DRG grouping; ICD-10-PCS is not used in mainland China. Medications are identified by Chinese generic and registered trade names; ATC codes are NOT implemented in Chinese EHRs, drug identification relies on the Chinese National Drug Code and local pharmacy item codes. Laboratory tests use local LIS codes with Chinese names, mapped to LOINC for reference only. TCM procedures and proprietary TCM medicines are captured directly in the HIS TCM module using institution-specific local codes. Abbreviations: AP = anteroposterior; ATC = Anatomical Therapeutic Chemical; BMI = body-mass index; COX-2 = cyclo-oxygenase-2; DRG = diagnosis-related group; EHR = electronic health record; ESWT = extracorporeal shock wave therapy; HIS = hospital information system; LIS = laboratory information system; LOINC = Logical Observation Identifiers Names and Codes; NHSA = National Healthcare Security Administration (China); NSAID = non-steroidal anti-inflammatory drug; PF = plantar fasciitis; PRP = platelet-rich plasma; RIS = radiology information system; TCM = traditional Chinese medicine; TENS = transcutaneous electrical nerve stimulation; UEBMI = Urban Employee Basic Medical Insurance; URRBMI = Urban-Rural Resident Basic Medical Insurance; YPID = Yao Pin Identifier.

**Supplementary Table SX. Week-by-week 12-week exercise protocol (both groups)**

| Week | Assessment | Goals | Manual therapy (hospital only) | Supervised exercises (sets × reps × freq) | Modalities | Remarks |
|---|---|---|---|---|---|---|
| 1 | NRS, FAAM, tenderness mapping, gait observation | Pain down-regulation; education | Soft-tissue mobilization of plantar fascia and gastrocnemius 10 min | Plantar fascia–specific stretching (DiGiovanni) 10 × 10 s, 3×/day; gastrocnemius/soleus wall stretch 3 × 30 s, 3×/day; isometric short-foot 10 × 5 s, 2×/day | Ice 15 min after activity; NSAIDs PRN; heel cushion | Education on load management, footwear, avoiding barefoot walking |
| 2 | NRS, adherence check | Reinforce stretching; begin isotonic intrinsics | Grade II talocrural and subtalar mobilization | Continue week 1; add towel curls 3 × 15, 1×/day; marble pick-up 2 × 10 | Same as week 1 | Confirm correct stretch technique (app video review in digital group) |
| 3 | NRS, FAAM-ADL | Introduce progressive loading | Cross-friction massage plantar fascia 5 min | Double-leg heel raises 3 × 12, 1×/day; continue week 2 exercises | Taper NSAIDs | If NRS not fallen ≥20% from baseline → Escalation A (Table S12) |
| 4 | NRS | Consolidate loading | Myofascial release of posterior compartment | Double-leg heel raises 3 × 15 with 3-s cadence; single-leg balance on firm surface 3 × 30 s | Optional ice post-exercise | Target ≥40% NRS reduction |

| Week | Assessment | Goals | Manual therapy (hospital only) | Supervised exercises (sets × reps × freq) | Modalities | Remarks |
|---|---|---|---|---|---|---|
| 5 | NRS, FAAM | High-load strength training (Rathleff) | Grade III mobilization if stiffness | Single-leg heel raise with towel under toes; 3-s concentric / 2-s hold / 3-s eccentric, 3 × 12 every other day; continue stretching | Discontinue ice unless flare | Load progressed by backpack mass; digital group uses in-app tempo metronome |
| 6 | NRS, adherence | Progress load | Joint mobilization as needed | 3 × 10 at higher load (4 × 8 if tolerated); balance on foam 3 × 30 s | — | Plateau rule: if no change ×2 consecutive weeks → Escalation B |
| 7 | NRS, FAAM-Sport | Functional loading | Neural glides if nerve-tension signs | Single-leg heel raises 4 × 8 heavy; step-ups 3 × 10 each leg; lateral band walks 3 × 12 | — | Introduce low-impact cardio (cycling, swim) |
| 8 | NRS, EQ-5D-5L | Dynamic stability | — | Single-leg hops (low) 3 × 8; wall sits 3 × 45 s; continue heel raises | — | Recovery check-point; if NRS ≤2 consider maintenance track |
| 9 | NRS, FAAM | Sport-specific / return | — | Bounding drills (light); skipping 3 × 30 s; running-specific calf loading; sport-specific movement rehearsal | — | Individualized by occupation/sport (Table S11) |

| Week | Assessment | Goals | Manual therapy (hospital only) | Supervised exercises (sets × reps × freq) | Modalities | Remarks |
|---|---|---|---|---|---|---|
| 10 | NRS, AOFAS | Return to full activity | — | Graded running program (walk–jog–run); plyometrics 3 × 6; continue strength 2×/week | — | Confirm absence of morning first-step pain >3 |
| 11 | NRS, WPAI | Maintenance | — | Strength 2×/week at highest tolerated load; stretching daily | — | Self-management plan finalized |
| 12 | NRS, FAAM, EQ-5D-5L, AOFAS | Discharge and relapse prevention | Discharge manual-therapy review | Home maintenance set (2×/week): heel raises, stretch, balance | — | Relapse-warning checklist; re-access criteria |

Both groups perform the same exercises in the same weekly order. The hospital group adds a manual-therapy column (in-person, 15–20 minutes per session, 2–3 sessions/week). The digital group receives manual-therapy content as a self-mobilization module via the Joymotion® app with weekly asynchronous therapist review.

**Supplementary Table SXI. Baseline tailoring rules (digital program)**

| Baseline characteristic | Tailoring rule |
| --- | --- |
| **BMI** | |
| Underweight (<18.5 kg/m$^2$) | Standard protocol with nutrition referral; monitor for fatigue. |
| Normal (18.5–23.9 kg/m$^2$) | Standard protocol. |
| Overweight (24–27.9 kg/m$^2$) | Standard protocol; reduce impact weeks 7–10; add cycling/elliptical cardio 3×/week. |
| Obese (≥28 kg/m$^2$) | Defer plyometrics to week 10; emphasize aquatic exercise weeks 1–4; weight-management module. |
| **Baseline pain severity** | |
| Mild (NRS 1–3) | Start at week 2 content; faster progression (1 week per step). |
| Moderate (NRS 4–6) | Standard 12-week progression. |
| Severe (NRS 7–10) | Extend weeks 1–2 content by 1 week; add pain-coping skills training module. |
| **Baseline function (FAAM–ADL)** | |
| FAAM-ADL ≥80 | Begin loading phase at week 3. |
| FAAM-ADL 50–79 | Standard progression. |
| FAAM-ADL <50 | Extend early phase; therapist check-in 2×/week. |
| **Age** | |
| <40 years | Standard. |
| 40–60 years | Standard; emphasize Achilles–soleus flexibility. |

| **Baseline characteristic** | **Tailoring rule** |
|---|---|
| >60 years | Replace single-leg hops with low-step-ups; balance progression on foam; halve high-load starting weight. |
| **Comorbidities** | |
| Diabetes | Daily foot inspection reminder; no ice >10 min; conservative load; HbA1c reviewed at enrollment. |
| Cardiovascular disease | RPE-guided cardio (target 11–13/20); defer plyometrics until medical clearance. |
| **Laterality** | |
| Unilateral disease | Standard; unaffected side for load calibration. |
| Bilateral disease | Volume split: alternate legs daily; total weekly volume per protocol. |
| **Occupation** | |
| Standing (≥6 h/day) | Micro-break stretch every 2 h; gel insole during shifts; gradual return. |
| Sedentary | Add hip-extension/glute activation; desk-side short-foot drills. |

**Supplementary Table SXIa. EQ-5D-5L utility trajectory (reconciled)**

| Time-point | Digital (n=128) | Hospital (n=256) | Between-group difference |
|---|---|---|---|
| Baseline | 0.742 (0.724 to 0.760) | 0.738 (0.726 to 0.750) | +0.004 (−0.018 to +0.026) |
| 4 weeks | 0.805 (0.784 to 0.826) | 0.791 (0.776 to 0.806) | +0.014 (−0.011 to +0.039) |
| 3 months | 0.862 (0.842 to 0.882) | 0.841 (0.826 to 0.856) | +0.021 (−0.004 to +0.046) |
| 6 months | 0.902 (0.884 to 0.920) | 0.887 (0.874 to 0.900) | +0.015 (−0.007 to +0.037) |
| 12 months | 0.918 (0.900 to 0.936) | 0.910 (0.898 to 0.922) | +0.008 (−0.014 to +0.030) |
| 12-month QALY (trapezoidal AUC) | 0.876 | 0.862 | +0.014 (+0.004 to +0.024) |

Reconciled utility values underpinning Figure 2D and the QALY calculation. Values are adjusted means with 95% CI in parentheses. Trapezoidal AUC over 12 months yields the ΔQALY of 0.014 used in the cost-utility analysis.

**Supplementary Table SXII. Regulation rules during follow-up**

| Trigger | Action |
|---|---|
| NRS reduction <50% of baseline by Week 3 (Escalation A) | Reduce load by one tier; add topical NSAID; pain-education push notification; therapist video call within 72 h. |
| NRS reduction <30% by Week 4 | Defer week-5 high-load introduction by 1 week; re-examine footwear and activity load; consider ultrasound re-imaging. |
| NRS reduction ≥50% by Week 4 (on-track) | Proceed to high-load strengthening (Rathleff, week 5). |
| NRS plateau from Week 6 (Escalation B) | Modify exercise selection; introduce eccentric-biased loading; consider gait-retraining module. |
| Worsening pain (NRS ≥2 points) at any time | Pause loading 3–5 days; week-1 stretching only; therapist reassessment; rule out stress fracture if indicated. |
| NRS ≤1 by Week 8 (full recovery) | Shift to maintenance track; check-ins fortnightly; encourage return to preferred activity. |
| ΔNRS <2 by Week 8 | Refer for ESWT per APTA 2023 CPG. |
| ΔNRS <2 by Week 12 + imaging-confirmed fasciopathy | Consider corticosteroid or PRP injection per shared decision. |
| Recurrence at 6–12 months | Re-enroll in abbreviated 6-week maintenance program. |
| Red flags (night pain, fever, swelling, neurovascular signs) | Immediate in-person evaluation; suspend exercise program. |
