# Supplementary material for: HOME-BASED DIGITAL EXERCISE VERSUS HOSPITAL PHYSIOTHERAPY FOR PLANTAR FASCIITIS: A PROPENSITY-SCORE-MATCHED RETROSPECTIVE COHORT STUDY WITH DUAL-PERSPECTIVE COST-UTILITY EVALUATION
Source: J Rehabil Med. 2026 Sep 2;58:45974. doi: 10.2340/jrm.v58.45974 (PMC13548366; doi:10.2340/jrm.v58.45974)
Supplement: Supplementary file 3 [file JRM-58-45974-s3.pdf]

Supplementary Materials

Abbreviations

AOFAS, American Orthopedic Foot and Ankle Society; APTA, American Physical Therapy Association; AUC, area under the curve; BMI, body mass index; CEAC, cost-effectiveness acceptability curve; CHEERS, Consolidated Health Economic Evaluation Reporting Standards; CI, confidence interval; CNNIC, China Internet Network Information Center; CNY, Chinese yuan; CPG, Clinical Practice Guideline; EHR, electronic health record; EQ-5D-5L, EuroQol five-dimension five-level; ESWT, extracorporeal shockwave therapy; FAAM, Foot and Ankle Ability Measure; GDP, gross domestic product; HbA1c, glycated hemoglobin; ICD-10, International Classification of Diseases 10th Revision; IPTW, inverse-probability-of-treatment weighting; MCID, minimal clinically important difference; MI, multiple imputation; MID, minimal important difference; NRS, numeric rating scale; NSAID, non-steroidal anti-inflammatory drug; PF, plantar fasciitis; PP, per-protocol; PROM, patient-reported outcome measure; PRP, platelet-rich plasma; PS, propensity score; PSA, probabilistic sensitivity analysis; QALY, quality-adjusted life-year; RCT, randomized controlled trial; RPE, rating of perceived exertion; SD, standard deviation; SMD, standardized mean difference; STROBE, Strengthening the Reporting of Observational Studies in Epidemiology; VAS, visual analog scale; WPAI, Work Productivity and Activity Impairment; WTP, willingness to pay.

Supplementary Methods

S1 Data extraction and quality assurance

SQL query extracted 68 candidate variables from the hospital Oracle-based clinical data warehouse. Two clinician reviewers re-abstracted 10% of charts; Cohen κ 0.91, intraclass correlation 0.96. Discrepancies adjudicated by a third reviewer. Primary outcome and all PROMs were collected through the hospital tablet-based intake station at each follow-up visit using a single harmonized form with identical wording in both groups. Dictionary in Supplementary Table S9.

S2 Non-inferiority margin derivation

Landorf and colleagues reported anchor-based minimal important differences for plantar heel pain of approximately 8 mm on a 100-mm visual analog scale for average pain (≈0.8 points on the 0–10 NRS scale by linear mapping) and approximately 19 mm for first-step pain (≈1.9 on the NRS scale) **[28]**. The prespecified primary margin of 1.3 points is the midpoint between these two anchors. This choice is more conservative than the first-step MID (1.9), more lenient than the average-pain MID (0.9), and reflects the dual relevance of both pain constructs for patients with plantar fasciitis. Non-inferiority was additionally declared against margins of 0.9 and 1.9 points as pre-specified sensitivity; all three margins yield the same conclusion (Table S3a).

S3 Sample-size justification

For a non-inferiority comparison of a continuous outcome with allocation ratio k=2 (two hospital patients per digital patient), the minimum number of digital patients is $n = (1 + 1/k)(z_{1-a} + z_{1-\beta})^2\sigma^2/\Delta^2$, with k·n hospital patients. With Δ=1.3 points on first-step NRS, expected SD σ=2.4, true difference 0, one-sided α=0.025 (z=1.960), and power 0.90 (z=1.282): $n = 1.5 \times (1.960+1.282)^2 \times 2.4^2 / 1.3^2 = 1.5 \times 10.51 \times 5.76 / 1.69 \approx 54$ digital and 108 hospital (162 in total). The realized matched sample (128:256) substantially exceeded this minimum. At the realized sample size the study had approximately 99% power against the 1.3-point margin and approximately 93% power against the more conservative 0.9-point margin; against the least-conservative 1.9-point margin the minimum requirement was 26 digital and 52 hospital.

S4 Propensity-score model specification

Logistic regression predicted digital-program receipt from 13 baseline covariates: age, sex, BMI, education at or above tertiary level, urban residence, insurance type (Urban Employee Basic Medical Insurance [reference], Urban-Rural Resident Basic Medical Insurance, or out-of-pocket), appointment made via mobile app or WeChat, e-prescription flag, distance to hospital, Charlson comorbidity index, baseline first-step morning NRS, prior PF episode, and type-2 diabetes mellitus. Insurance type was modeled as a single categorical covariate with three levels. Continuous covariates (age, BMI, distance to hospital) entered as restricted cubic splines with three knots at the 10th, 50th, and 90th percentiles. Discrimination (c-statistic 0.78); calibration (Hosmer–Lemeshow p=0.42); 1:2 greedy nearest-

neighbor matching without replacement with caliper 0.2 × SD of the logit; balance evaluated by SMD and variance ratio; SMD threshold 0.10.

S5 Mixed-effects primary model

$Y_{ijk} = \beta 0 + \beta 1 \cdot Treatment_{jk} + \beta 2 \cdot Y0_{jk} + \Sigma\beta_m \cdot X_{mjk} + (\text{time and treatment} \times \text{time terms}) + u_k + v_{jk} + \varepsilon_{ijk}$, where Y_{ijk} is the outcome at follow-up time i for patient j in matched set k, $Y0_{jk}$ the baseline value, X_{mjk} the 13-covariate vector, u_k a matched-set random intercept, v_{jk} a patient-level random intercept nested within matched set, and ε_{ijk} the residual. The design is hierarchical at three levels, because repeated measurements are nested within patients and patients are nested within 1:2 matched sets, so random effects are required at both the matched-set and the patient level. Fitting u_k alone would impose an exchangeable (compound-symmetry) correlation across all observations belonging to a matched set, so that the 3-month value of one patient and the 12-month value of a different patient in the same set would be assumed as strongly correlated as two measurements taken on the same patient. That structure is implausible for longitudinal data and produces standard errors, and hence confidence intervals and p-values, that are not valid. Including v_{jk} separates the within-patient (repeated-measures) correlation from the between-patient correlation induced by matching, while retaining u_k preserves the pairing created by the design. Variance components for u_k and v_{jk}, with the corresponding intraclass correlations, are reported in Supplementary Table S5b. Follow-up time entered as a categorical factor (3, 6, and 12 months), imposing no functional-form constraint on the trajectory shape, with a treatment × time interaction. Denominator degrees of freedom by Kenward–Roger, which is appropriate given the modest number of matched sets. Three pre-specified sensitivity or confirmatory models were fitted: (a) a parsimonious model retaining only baseline score as a covariate; (b) a model adding a patient-level random slope for time, $v1_{jk} \cdot time_{ijk}$, within the nested structure; and (c) a confirmatory matched-set analysis of covariance restricted to the 3-month primary endpoint, $Y_{jk} = \beta 0 + \beta 1 \cdot Treatment_{jk} + \beta 2 \cdot Y0_{jk} + \Sigma\beta_m \cdot X_{mjk} + u_k + \varepsilon_{jk}$, in which each patient contributes a single observation, the time factor and the treatment × time interaction are omitted, and matched-set clustering is the only remaining source of non-independence.

S6 E-value computation

Continuous-outcome E-value per VanderWeele–Ding: RR ≈ exp(0.91 × d), where d = adjusted mean difference / SD. We report E-values under two SD choices: (i) pooled baseline SD of 1.6, giving d=0.26, RR≈1.27, E=1.85 (lower CI 1.22); (ii) within-group residual SD of 1.1, giving d=0.38, RR≈1.41, E=2.14 (lower CI 1.32). The pooled baseline choice is more conservative; both are reported for transparency. The 0.91 transformation constant derives from Chinn 2000 for converting standardized mean differences into log-odds-ratios for approximately symmetric continuous outcomes.

S7 Missing data and pattern-mixture δ-shift

MI addressed missing post-baseline outcome data. Missing 3-, 6-, and 12-month NRS and patient-reported outcome values (arising when a follow-up visit was not attended) were imputed; fully observed baseline covariates and baseline scores were used as predictors and were not themselves imputed. MI with chained equations generated m=20 completed datasets (predictive mean matching for continuous, logistic regression for binary); imputation models included treatment, observed outcomes at other time-points, and all covariates. The imputation was applied to all 384 matched patients, and results were pooled across the 20 datasets by Rubin's rules. Pattern-mixture: imputed values in the digital group shifted upward by $\delta \in \{0.5, 1.0, 1.5, 2.0\}$ NRS to simulate informative dropout. Convergence verified by trace plots and between-chain mixing.

S8 Economic analysis details

Costs in 2024 CNY. Direct costs: hospital billing, co-payments, transportation, informal caregiver time. Indirect costs: WPAI absenteeism and presenteeism valued at provincial mean wage ¥12,850/month. Therapist asynchronous-review time in the digital group included in program tariff and payer-perspective cost. No discounting (12-month horizon). QALYs by trapezoidal AUC over EQ-5D-5L utilities at baseline, 4 weeks, 3, 6, and 12 months, Luo 2017 Chinese tariff. Probabilistic sensitivity: 1000 bootstrap replicates with matched-pair cluster resampling, fixed seed. WTP threshold ¥157,300/QALY = 1.76 × 2023 GDP per capita. CEACs also computed at 1× GDP and 3× GDP.

S9 Calendar-time COVID-19 sensitivity

The 2022–2024 study window covers lockdown (pre-January 2023), reopening (Q1 2023), and post-reopening periods. Enrollment quarter included as PS-model covariate. Sensitivity analysis restricted to post-January 2023 enrollments (n=263 matched) gave adjusted mean difference −0.38 (95% CI −0.82 to +0.06), substantively similar to primary estimate (−0.42).

S10 Tipping-point analysis for differential outcome measurement

A quantitative bias analysis was performed to assess how large a differential measurement error between groups would be required to nullify the primary finding. A uniform shift of at least −0.45 NRS applied to all hospital-group 3-month measurements would be required to move the upper 97.5% confidence limit above zero.

S11 Bias mitigation map

Threat to internal validity	Mitigation applied
Confounding by indication	PS matching on 13 covariates; mixed-effects regression within matched cohort; IPTW on full cohort; E-value.
Unmeasured digital-adoption confounders	Acknowledged in Limitations; directional discussion; E-value bounds single-confounder effect.
Selection bias (12-week record eligibility)	IPTW sensitivity on full cohort including partial-record patients.
Information bias (ascertainment asymmetry)	Harmonized tablet-based PROM instrument; tipping-point analysis.
Attrition bias	MI with chained equations (m=20); pattern-mixture δ-shift.
Calendar-time (COVID-19) confounding	Enrollment quarter in PS model; post-January 2023 sensitivity analysis.

Threat to internal validity	Mitigation applied
Immortal-time bias	Not applicable (fixed 12-week window from index visit).
Therapist clustering (hospital group)	Acknowledged as a limitation; therapist-level clustering was not separately modeled beyond the matched-pair random effect.

S12 Software

R 4.3.2: MatchIt 4.5.5, cobalt 4.5.2, lme4 1.1-35, lmerTest 3.1-3, pbkrtest 0.5.2, mice 3.16, boot 1.3-30, EValue 4.1.3, ggplot2 3.5. Nested random effects were fitted with lme4 using the (1 | matched_set/patient) specification; Kenward–Roger denominator degrees of freedom were obtained via pbkrtest.
